# Transcriptome and association mapping revealed functional genes respond to drought stress in *Populus*

**DOI:** 10.3389/fpls.2022.829888

**Published:** 2022-07-29

**Authors:** Fangyuan Song, Jiaxuan Zhou, Mingyang Quan, Liang Xiao, Wenjie Lu, Shitong Qin, Yuanyuan Fang, Dan Wang, Peng Li, Qingzhang Du, Yousry A. El-Kassaby, Deqiang Zhang

**Affiliations:** ^1^National Engineering Laboratory for Tree Breeding, College of Biological Sciences and Technology, Beijing Forestry University, Beijing, China; ^2^Key Laboratory of Genetics and Breeding in Forest Trees and Ornamental Plants, Ministry of Education, College of Biological Sciences and Technology, Beijing Forestry University, Beijing, China; ^3^Department of Forest and Conservation Sciences, Faculty of Forestry, Forest Sciences Centre, University of British Columbia, Vancouver, BC, Canada

**Keywords:** association genetics, co-expression, eQTN, epistasis, drought tolerance, *Populus*

## Abstract

Drought frequency and severity are exacerbated by global climate change, which could compromise forest ecosystems. However, there have been minimal efforts to systematically investigate the genetic basis of the response to drought stress in perennial trees. Here, we implemented a systems genetics approach that combines co-expression analysis, association genetics, and expression quantitative trait nucleotide (eQTN) mapping to construct an allelic genetic regulatory network comprising four key regulators (*PtoeIF-2B*, *PtoABF3*, *PtoPSB33*, and *PtoLHCA4*) under drought stress conditions. Furthermore, Hap_01PtoeIF-2B, a superior haplotype associated with the net photosynthesis, was revealed through allelic frequency and haplotype analysis. In total, 75 candidate genes related to drought stress were identified through transcriptome analyses of five *Populus* cultivars (*P. tremula × P. alba*, *P. nigra*, *P. simonii*, *P. trichocarpa*, and *P. tomentosa*). Through association mapping, we detected 92 unique SNPs from 38 genes and 104 epistatic gene pairs that were associated with six drought-related traits by association mapping. eQTN mapping unravels drought stress-related gene loci that were significantly associated with the expression levels of candidate genes for drought stress. In summary, we have developed an integrated strategy for dissecting a complex genetic network, which facilitates an integrated population genomics approach that can assess the effects of environmental threats.

## Introduction

Drought is an inevitable and recurring feature of global climate change, it is increasing in frequency and intensity. Forest trees constitute ~45% of global terrestrial carbon stocks and have key roles in ecosystem stability (Rogers et al., 2018). Extreme drought is greatly harmful to forest trees, it causes substantial productivity losses, affects ecosystem security, and threatens human survival (Vitasse et al., 2019). Thus, there is a need to explore the genetic architecture and regulatory mechanisms of drought stress in forest tree populations. Drought tolerance is a complex trait that involves several mechanisms, including escape, avoidance, and tolerance (Gupta et al., 2020). Plants drought stress mechanisms are related to hydraulic signals, reactive antioxidants, osmotic regulation, and phytohormone movements (Attipalli et al., 2004; Ahmad et al., 2010). Under drought stress, rapid expression of the *P5CS* gene in barley led to proline accumulation (Frimpong et al., 2021). Additionally, the over-expression of *PeCHYR1* (CHY-type/CTCHY-type/RING-type zinc finger protein) significantly improved drought tolerance in poplar trees by enhancing hyperoxide production and reducing the stomatal aperture (He et al., 2018). However, naturally occurring drought stress variation and the effects of drought stress adaptation at the species and population levels have not been systematically investigated.

Association genetics using molecular marker-based technologies, enables decryption of the genetic basis of phenotypic variation in forest trees. Specifically, population genomics promotes genetic improvement of drought tolerance and the development of diagnostic tools for the conservation and management of forest tree natural populations (Neale and Kremer, 2011). Additionally, association mapping is a widely used approach to investigate the allelic variants that underpin complex traits, it is particularly powerful in forest trees because of the high levels of nucleotide diversity and low linkage disequilibrium in perennial woody plants (Wegrzyn et al., 2010; Beaulieu et al., 2011; Guerra et al., 2013). In particular, association studies concerning additive, dominant and epistatic gene effects have provided insights into the genetic architecture that underlies plant phenotypic variation (Du et al., 2015; Deng et al., 2017). This strategy enables dissection of the genetic effects of multi-gene networks in *Populus*, allowing clarification of the genetic regulation of complex traits in trees (Quan et al., 2019). Expression quantitative trait nucleotide (eQTN) mapping, defined as associations between SNPs and expression level of candidate genes, is used to decipher the allelic variations that contribute to phenotypes at the transcriptional level, thus facilitating investigation of the effects of candidate genes (Lu et al., 2021). Co-expression network analysis allows the integration of transcriptome data types and clustering of genes with correlated expression patterns into co-expression modules, these capabilities permit exploration of the functional connections between candidate genes involved in the same or shared biological pathways (Serin et al., 2016). Thus, the combination of co-expression network analysis, association genetics, and eQTN mapping will provide insights into the genetic architecture that underlies the response of trees to drought stress.

*Populus* is a major fast-growing plantation tree genus used for bioenergy, timber, and pulp manufacturing; it also is an excellent model system of long-lived forest trees for biological studies related to environmental changes (Jansson and Douglas, 2007; Lu et al., 2021; Zhao et al., 2021). *Populus* comprises >30 species and is geographically distributed throughout the northern hemisphere (Taylor, 2002). However, most fast-growing poplar varieties have poor drought stress tolerance (Tuskan et al., 2006; Lüttschwager et al., 2015). The construction of a systematic network and identification of candidate genes related to the drought stress response would improve our understanding of drought stress in *Populus*. Here, we firstly used transcriptome data of five representative poplar species (*P. tremula × P. alba*, *P. nigra*, *P. simonii*, *Populus trichocarpa*, and *P. tomentosa*) to identify differentially expressed genes (DEGs) under drought conditions. Through weighted gene co-expression network analysis (WGCNA), and based on DEGs, we detected three important modules and 75 candidate genes related to drought stress. Next, we performed association mapping to identify the significant associated loci and genes for six drought-responsive traits in an association population of 300 *P. tomentosa* accessions under drought stress. Based on these findings, we proposed the genetic networks in the drought stress response pathway, which will be useful for molecular marker-assisted breeding of drought tolerant individuals in poplar. Expression quantitative trait nucleotide (eQTN) mapping combined with the analysis of six drought-responsive traits aided our interpretation of candidate genes related to drought stress. Our method will enable the exploration of the candidate genes related to drought tolerance for molecular marker-assisted selection (MAS) of drought-tolerant varieties of poplar.

## Materials and methods

### Plant materials and drought stress treatment

The association population consisted of 300, one-year-old *P. tomentosa* accessions with three ramets of each genotype, which were asexually propagated *via* root segments in 2018 in Guan Xian County, Shandong Province, China (36°23′N, 115°47′E); this area represents most of the species’ natural distribution range. The distribution of these individuals was divided into southern (S, *n* = 94), northwestern (NW, *n* = 108), and northeastern (NE, *n* = 108) geographical regions (Huang, 1992). All individuals were well-watered by an automatic irrigation system three times per week and subjected to a well-watered (WW) period for 20 days to ensure their root development. Water deficit (WD) treatment began when leaf 6 (L6) was initiated on the apex, according to visual inspection (Boyes, 2001). The drought stress treatment was as follows: (1) 20 days well-watered (WW); (2) followed by 30 days water deficit (WD) period until 70% of the leaves became wilted and yellow; and (3) then, a re-watering (RW) period for 20 days three times per week (Supplementary Figure S1). The volumetric soil water content (SWC) was measured using a model 4,300 neutron attenuation soil moisture meter and used to evaluate the degree of drought (the soil water contents were ~10% for WD and 40% for both WW and RW) (Grote et al., 2010). The daily mean minimum and maximum temperatures from WW to WD were 32.3°C and 37.6°C, respectively; these minimum and maximum temperatures were 34.2°C and 39.0°C, respectively, from WD to RW. Plants were exposed to the ambient mean relative air humidity (67.5%), with minimal precipitation. Functional leaves (i.e., the fourth to sixth leaves from the top of the stem) were collected from three biological replications separately from the 300 individuals, then three technical replications of each sample were conducted separately. The same experiment was conducted at well-watered (WW) condition, water deficit (WD) condition and re-watering (RW) condition, separately. The samples were immediately immersed in liquid nitrogen and stored at −80°C prior to vacuum freeze-drying. The leaf materials were used for subsequent drought stress index measurement.

### Phenotype analysis

Photosynthesis, proline content (PRO), and catalase activity (CAT) are highly sensitive to changes in environmental factors, including drought stress. We measured six drought stress-related traits under water deficit (WD) and well-watered (WW) conditions. The photosynthetic traits were net photosynthesis (Pn), stomatal conductance (Cond), transpiration rate (Trmmol), and relative chlorophyll content (Chl). The proline content (PRO) and catalase activity (CAT) were also measured. The phenotypic variation of the traits is provided in Supplementary Table S1.

Photosynthetic traits were measured from fully expanded leaves (three functional leaves, the top fourth to sixth leaves) using a portable photosynthesis system (LI-6400, LI-COR, Lincoln, NE, Unites States) in accordance with the manufacturer’s instructions. Each genotype was measured on sunny days between 9:00 and 11:30 a.m. under a fixed light intensity of 1,200 μmol m^−2^ s^−1^ during the drought treatment. All measurements were performed using three replicates per individual genotype. Next, we used a portable chlorophyll meter (SPAD-502, Konica-Minolta, Japan) to measure the leaf chlorophyll concentration, which is presented as the SPAD value. For each leaf, the chlorophyll content was estimated as the mean of 10 SPAD values at different positions of the leaf middle section excluding the leaf midrib.

After the photosynthetic characteristics had been measured, the same functional leaves were immediately collected from the 300 accessions (separately from three ramets of each accession) and frozen in liquid nitrogen for PRO and CAT measurement. Proline content was extracted from 1.0 g of fresh leaves using 10 ml of 3% sulfosalicylic acid at 100°C for 10 min. A 4-ml aliquot of the extract was then mixed with 4 ml of ninhydrin reagent containing glacial acetic acid, then incubated at 95°C for 30 min. The reaction mixture was quickly cooled with running tap water. The colored reaction product was extracted with 8 ml of toluene, and the absorbance of the toluene phase was measured at 520 nm using a spectrophotometer (Shimadzu, Model UV 1800, Kyoto, Japan). To determine CAT activity in leaf extracts, 30 μl of extract were added to 50 mM K-phosphate buffer (pH 7.0) and 2% H_2_O_2_ for a total volume of 3 ml. Enzyme activity was calculated based on the absorbance at 240 nm recorded for 2 min using a spectrophotometer (see above).

Coefficient of variation (CV) values defined as the ratio of the standard deviation (SD) to the mean of each drought stress-related trait in the population, were independently calculated using the mean of the biological replicates of the untransformed drought stress-related traits data. The Pearson correlation coefficient (*r*) for each drought stress-related trait pair was calculated using the R package psych (Revelle, 2017).

### Transcriptome data processing

Fully expanded leaves of 10 *P. tomentosa* genotypes, which covered three different regions were collected at WW (well-watered) and WD (water deficit) time points, respectively. The collected leaves were immediately immersed in liquid nitrogen, and stored at −80°C. Total RNA was extracted using a Qiagen RNeasy Kit (Qiagen China, Shanghai, China), in accordance with the manufacturer’s instructions. In addition, DNase digestion was performed using an RNase Free DNase Kit (Qiagen). Detailed descriptions of the methods used for processing transcriptome data are provided in the supplemental materials (Supplementary Methods S1; Xiao et al., 2019).

*Populus tremula × P. alba*, *P. nigra*, *P. simonii*, and *P. trichocarpa* transcriptome data under different drought conditions were obtained from the NCBI SRA database (Gene Expression Omnibus, http://www.ncbi.nlm.nih.gov/sra), and saved in FASTQ format using the SRA Toolkit. The quality control method is described in Method S1. In total, we obtained an expression data set composed of 58 RNA-seq samples and 27,644 genes (Supplementary Tables S3, S4). Transcript expression level was normalized by calculating the Z-score based on fragments per kilobase of transcript per million fragments (FPKM) method (Supplementary Table S4).

The linear model LIMMA package in Bioconductor^1^ was used to perform differential gene expression analysis for the five species (Ritchie et al., 2015; genes with |Log_2_ (fold-change)| > 1 (*p* < 0.05) for DEGs). Gene ontology (GO) analysis was performed *via* AgriGO,^2^ based on *P. trichocarpa* v3.0 annotation. Pathway enrichment analysis was conducted using the Kyoto Encyclopedia of Genes and Genomes (KEGG) database and a hypergeometric statistical test.^3^

### Weighted gene co-expression network analysis analyses of DEGs

Weighted gene co-expression network analysis to identify key modules and hub genes is increasingly used in bioinformatics analyses in various biological contexts (Smita et al., 2013). An expression matrix based on 1,236 genes differentially expressed in more than three species in response to drought stress were used to construct a weighted gene co-expression network using the WGCNA package (Langfelder and Horvath, 2008). Weighted gene co-expression network analysis network construction and module detection were conducted using an unsigned type of topological overlap matrix (TOM), a power β of 8, a minimal module size of 30, and a branch merge cut height of 0.25. The adjacency matrix dissimilarity was 0.2. We then obtained several key network properties such as the edge weight and node connectivity. To identify the hub genes of a module, genes with edge weight ≥ 0.5 and the node connectivity ≥10 in the network were considered to be hub genes. Then Cytoscape (v.2.8.3) was used to visualize the correlation relationships between specified genes (Cline et al., 2007).

### Reverse transcription-quantitative polymerase chain reaction

Five genes were selected for validation of their expression profiles in 10 individuals with different genotypes using RT-qPCR. Three leaves (the fourth to sixth from the top of the stem) were collected from 10 one-year-old *P. tomentosa* seedlings and immediately immersed in liquid nitrogen. Total RNA was extracted from each leaf and reverse transcribed into cDNA using the Reverse Transcription System (Promega Corporation, Madison, WI, United States). Reverse transcription-quantitative PCR (RT-qPCR) was performed on the 7,500 Fast Real-Time PCR System using SYBR Premix Ex Taq (TaKaRa, Dalian, China), in accordance with the manufacturer’s protocol. Specific primer pairs for each gene were designed using Primer-BLAST software (Ye et al., 2012; Supplementary Table S8). All reactions were performed with triplicate technical and triplicate biological repetitions, with actin (EF145577) as the internal control, in accordance with the PCR program described by Xiao et al. (2019).

### Genome re*-*sequencing and SNP/InDel calling of *Populus tomentosa* association population

We used a Plant DNeasy Mini kit (Qiagen, Shanghai, China) to isolate the total genomic DNA of the 300 *P. tomentosa* unrelated individuals, in accordance with the manufacturer’s instructions. Total genomic DNA was re-sequenced at a depth > 15 × (raw data) using the Illumina GA2 sequencing platform. The clean reads were mapped to the *P. trichocarpa* reference genome v.3.0; they were used to perform SNP calling. SNP calling as described by Xiao et al. (2019). VCFtools was used to extract the gene-derived biallelic SNPs/InDels within the genes, including their 1,000 bp upstream and 1,000 bp downstream sequences. Finally, 5,553 SNPs of 75 candidate genes from the 300 accessions were used for association analysis (Supplementary Table S12).

### Association analysis

#### Single SNP*-*based association

The mixed linear model (MLM) in Tassel 5.0 was used to test the statistical associations between SNPs and the drought stress-related traits which were normalized based on the Z-score (Bradbury et al., 2007), after accounting for the population structure (Q) and pairwise kinship coefficients (K). The K matrix was derived by SPAGeDiv1.3 (Hardy and Vekemans, 2002) and the Q matrix was determined *via* STRUCTURE v.2.3.4 based on significant sub-populations (*k* = 3) (Evanno et al., 2005). The QVALUE package in R was used to correct for multiple testing with the positive false discovery rate (FDR) method (Storey, 2003). SNPs were considered significantly associated at *p <* 0.001 and *q* < 0.05 were identified. Manhattan plots and Q-Q plots were created using the qqman package in R v.3.0.2 (Mukrimin et al., 2018). Haplotype analysis was performed through Haploview v.4.2 software with default parameters (Barrett et al., 2005). Superior haplotypes were identified in accordance with the method established by Lv et al. (2021).

#### Multi*-*SNP epistasis association analysis

The EPISNP package in the epiSNP v.4.2 software suite was used to analyze epistatic effects (Ma et al., 2008). The SNP–SNP interaction effect with phenotypic traits was partitioned into four epistatic effects based on the extended Kempthorne model: additive × additive, additive × dominant, dominant × additive, and dominant × dominant epistatic effects. The significance level was defined as *p <* 0.001. Only the SNPs that demonstrated significance (*p* < 0.01) in SNP-based association mapping were used for epistasis analysis. A multifactor dimensionality reduction (MDR) algorithm was conducted to investigate the genotype combination effects in our studies (Hahn et al., 2003).

### eQTN mapping

eQTN mapping was performed using Tassel v.5.0 software, with a method identical to the SNP-based association analysis. eQTNs were considered significantly associated at *p* < 0.001 and *q* < 0.05. RNA-seq was used to measure the transcript levels of genes from the functional leaves of the 300 *P. tomentosa* individuals. RNA library construction and sequencing were performed by Beijing Biomarker Technology Cooperation (Beijing, China). The FPKM values (i.e., gene expression levels) were calculated as described in Supplementary Methods S1. Gene expression traits with missing data >20% and expression levels <0.1 (FPKM < 0.1) in >95% of the 300 individuals were removed. The detected eQTNs located in the 10-kb window around the expressed gene were defined as cis-eQTNs, and the remaining eQTNs were regarded as trans-eQTNs.

### Sequence analysis and phylogenetic tree construction

Amino acid sequences were obtained from the NCBI database,^4^ which were aligned and used to infer their phylogenetic relationships. Multiple sequence alignment was performed with MEGA v.6.0. The phylogenetic tree was constructed using MEGA v.6.0 with the neighbor-joining (NJ) algorithm and Kimura two-parameter model. Genetic distance was calculated using sequence pairwise alignments. The reliability of nodes on the neighbor-joining tree was estimated using a bootstrap analysis with 1,000 replicates.

## Results

### Construction of co*-*expression networks in *Populus* under drought stress

Comparative analyses were conducted for the five poplar species to identify drought-responsive genes. 1,236 drought-responsive DEGs (i.e., genes differentially expressed in >3 species in response to drought stress) were identified among the five species; these comprised 261 (35.23%) up- and 975 (64.77%) down-regulated genes (Supplementary Table S4). GO analysis of the 1,236 DEGs revealed the enrichment of 78 significant terms (*p* < 0.05) related to biological processes such as photosynthesis, multiple hormone-mediated regulations, and energy metabolism (Supplementary Table S6). KEGG analysis indicated that the DEGs were enriched in pathways such as photosynthesis and oxidative phosphorylation (Supplementary Table S7). Transcriptome analysis suggested that photosynthesis was the process most susceptible process in response to drought stress. We validated the expression levels of five randomly selected DEGs by RT-qPCR (Supplementary Figure S2). The expression patterns of the five genes according to RT-qPCR were similar to the patterns identified by RNA-seq, thereby validating the RNA-seq results.

**Figure 1:**
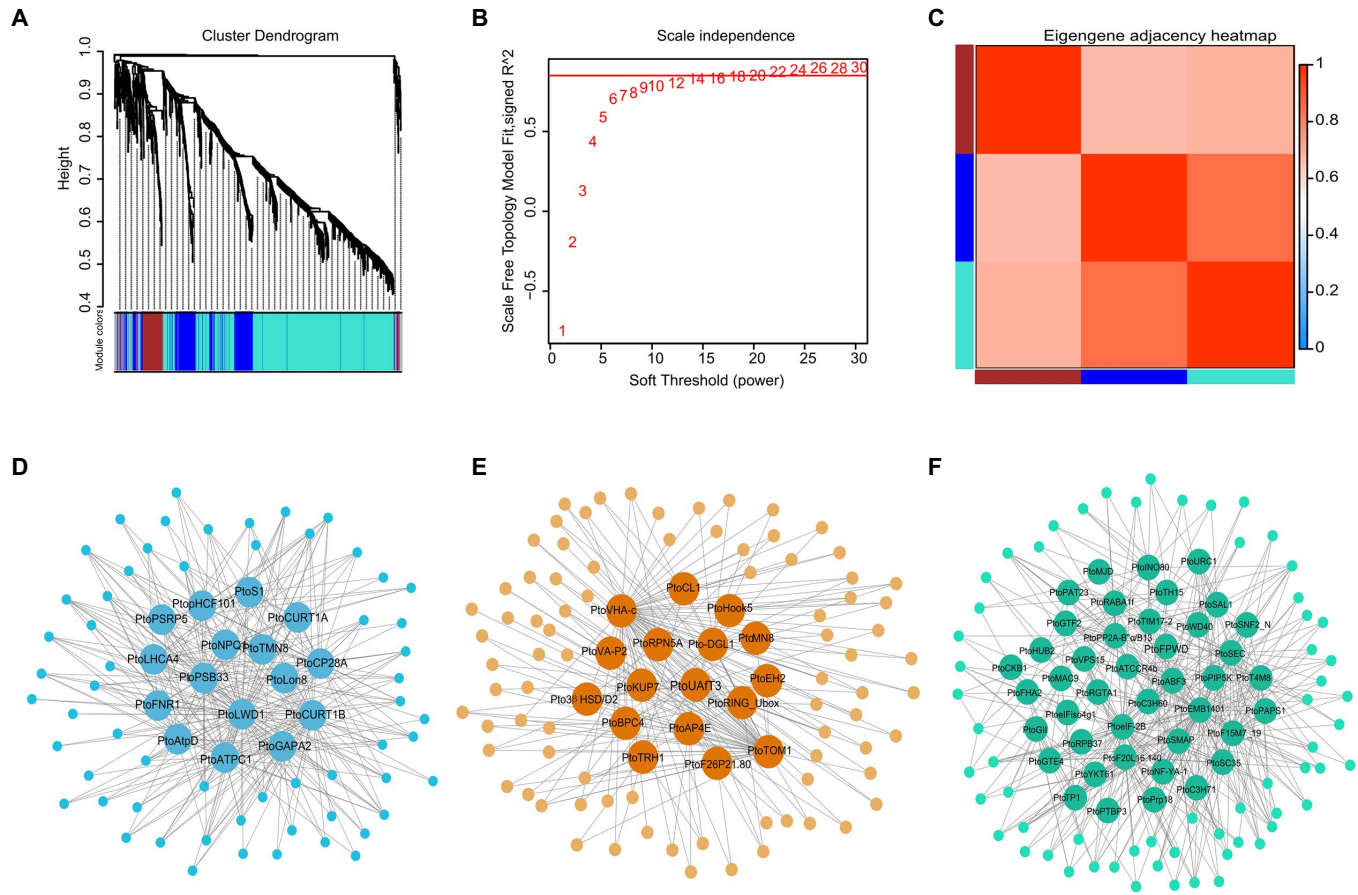
Co-expression network of differentially expressed genes (DEGs) in five poplar species. **(A–C)** Clustering dendrograms of gene expression patterns. Dissimilarity was based on topological overlap, along with assigned module colors. The three co-expression modules are shown in different colors. **(D–F)** Network relationship among the modules. In total, 16, 17, and 42 hub genes included in the blue, brown, and turquoise modules, respectively. Hub genes with edge weight ≥0.5 and node connectivity ≥10 were used to construct the network in Cytoscape.

A weighted co-expression network was constructed using 1,236 DEGs in the five-poplar species. The blue, brown, and turquoise modules contained 376, 168, and 692 genes, respectively, these results implied highly similar expression patterns among candidate genes (see the dendrogram in Figures 1A–F; Supplementary Table S5). Of the three modules, 75 hub genes with edge weight ≥ 0.5 and node connectivity ≥10 were selected. There were 16, 17, and 42 hub genes included in the blue, brown, and turquoise modules, respectively (Figures 1D–F; Supplementary Table S9). Among the 75 hub genes, most were known to be involved in drought stress. For example, the defense response gene *PtoRGP* (reduction in growth and productivity) regulates cellular processes that are involved in growth and abiotic stress responses (Lee et al., 2014); *PtoVHA-c* (encodes hydrolysis of the V-ATPase c subunit) confers stress tolerance through enhancing superoxide dismutase and peroxidase activities under drought stress (Cheng et al., 2013), and *PtoHDA15* (histone deacetylase) inhibits abscisic acid (ABA) signaling genes (Liu et al., 2013). Additionally, several novel high-degree hub gene signatures were identified in our analysis, such as *PtoRPN5A* (26S proteasome regulatory protein) and *PtoNRP1* (nodulin-related protein 1), a DNA-binding protein. To assess the roles of these 75 hubs in the network, we used these hub genes from each module and conducted a GO analysis. The genes were significantly enriched in hormone-mediated biosynthesis or antioxidant process (hydrogen peroxide) and photosynthetic components (cellular component; Supplementary Figure S3; Supplementary Table S6), which is consistent with the notion that hub genes typically play roles in the integration of other genes within a module (Ravasz, 2002).

### Allelic variation significantly associated with drought stress-related traits in *Populus tomentosa*

To further explore the genetic effects of candidate genes for drought stress-related traits in the co-expression network, six drought stress-related traits (Pn, Cond, Trmmol, Chl, PRO, and CAT) in functional leaves were measured in the 300 *P. tomentosa* individuals under WD conditions. Four photosynthetic traits were decreased under drought stress, while PRO, and CAT were increased (Supplementary Table S1). All six drought stress-related traits exhibited high genetic variation, with the coefficient of variation (CV) values ranging from 0.15 (Cond) to 7.59 (PRO; Supplementary Table S1). Estimates of heritability showed that four of six drought stress-related traits were with the broad-sense heritability (*H*^2^) > 0.6 (Supplementary Table S1); thus, illustrated they were presumed to be controlled by genomic variants. Pearson correlation analysis showed that traits within the same category were often closely correlated (Supplementary Table S2). These results indicated that the association population possessed significant genetic variability and could be used for population genetics analysis of the response to drought stress.

We conducted an association analysis concerning the genetic associations of 5,553 SNPs in 75 candidate genes with six drought stress-related traits (Supplementary Figure S4; Supplementary Table S12). The model identified 92 unique SNPs from 38 genes that showed significant associations with the six drought-related traits (*p* < 0.001, *q* < 0.05); the mean explained phenotypic variation (*R*^2^) was 9.40% (range: 0.13%–28.24%; Supplementary Table S13). Of these associations, 11 showed a combination of additive and dominant effects (Supplementary Table S13). Furthermore, three SNPs were simultaneously associated with two traits, indicating that they had pleiotropic effects on different drought related traits. For example, PtoLHCA4_SNP4 (T/A), located in the 3’UTR region of *PtoLHCA4* (encodes chlorophyll a-b binding protein), was simultaneously associated with Chl (*R*^2^ = 21.11%), and PRO (*R*^2^ = 9.68%). PtoABF3_SNP31 (A/G) was simultaneously associated with Cond (*R*^2^ = 23.27%), and Pn (*R*^2^ = 8.02%). Finally, PtoWD40_SNP25 (T/A), located in the intron region of *PtoWD40* (transducin family protein/WD-40 repeat family protein), was associated with Cond (*R*^2^ = 13.32%), and Pn (*R*^2^ = 7.49%; Supplementary Table S13).

**Figure 2:**
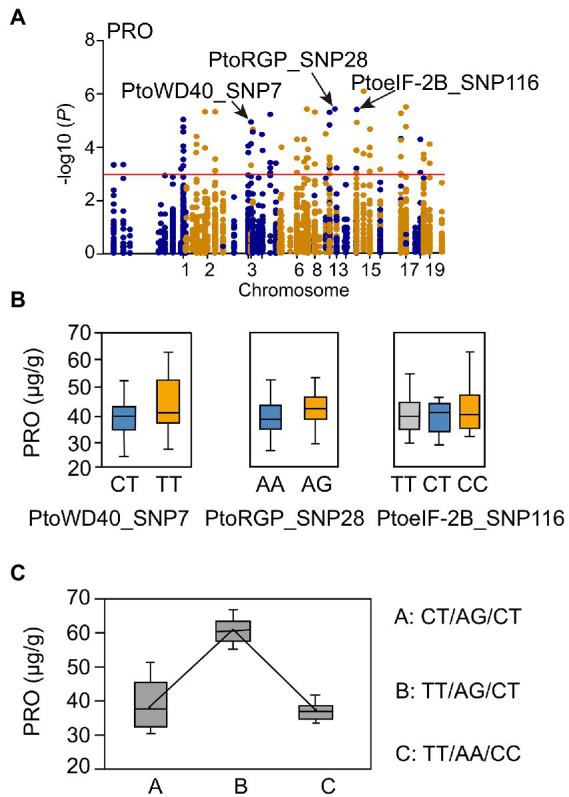
Proposed multi*-*SNP*-*based genotypic combinations for proline content (PRO). **(A)** Manhattan plot for PRO marked with candidate genes. **(B)** Genotypic effects of causal SNPs for PRO. **(C)** Three possible genotypic combinations with a frequency of ≥5% from the three allelic variations, and the genotypic combination effects for proline content in the *P. tomentosa* association population. SNPs in each genotypic combination were ordered according to **(B)**.

We also detected multiple SNPs that were associated with the same trait (Supplementary Table S13). Notably, there were 44 SNPs associated with PRO in 23 annotated genes. Genes in the turquoise module were mainly enriched in photosynthetic components (Supplementary Figure S3; Supplementary Table S6). Three candidate genes (*PtoWD40*, *PtoRGP*, and *PtoeIF-2B*) in the turquoise module were selected (Supplementary Tables S9, S11). The SNPs (PtoWD40_SNP7, PtoRGP_SNP28, and PtoeIF-2B_SNP116) were significantly associated with PRO (Figure 2A; Supplementary Table S13). Distinct genotypes of the three SNPs contributed differently to PRO; there were three possible common genotypic combinations (frequency > 5%, *p* < 0.01) for PRO (Figures 2B,C). The genotypic combinations of three SNPs led to PRO phenotypic differences, in which TT-AG-CT and TT-AA-CC combinations represented the maximum (63.20 μg/g) and minimum (37.44 μg/g) phenotypic values (Figure 2C).

### Pairwise epistasis of candidate loci revealed complex genetic networks under drought stress

**Figure 3:**
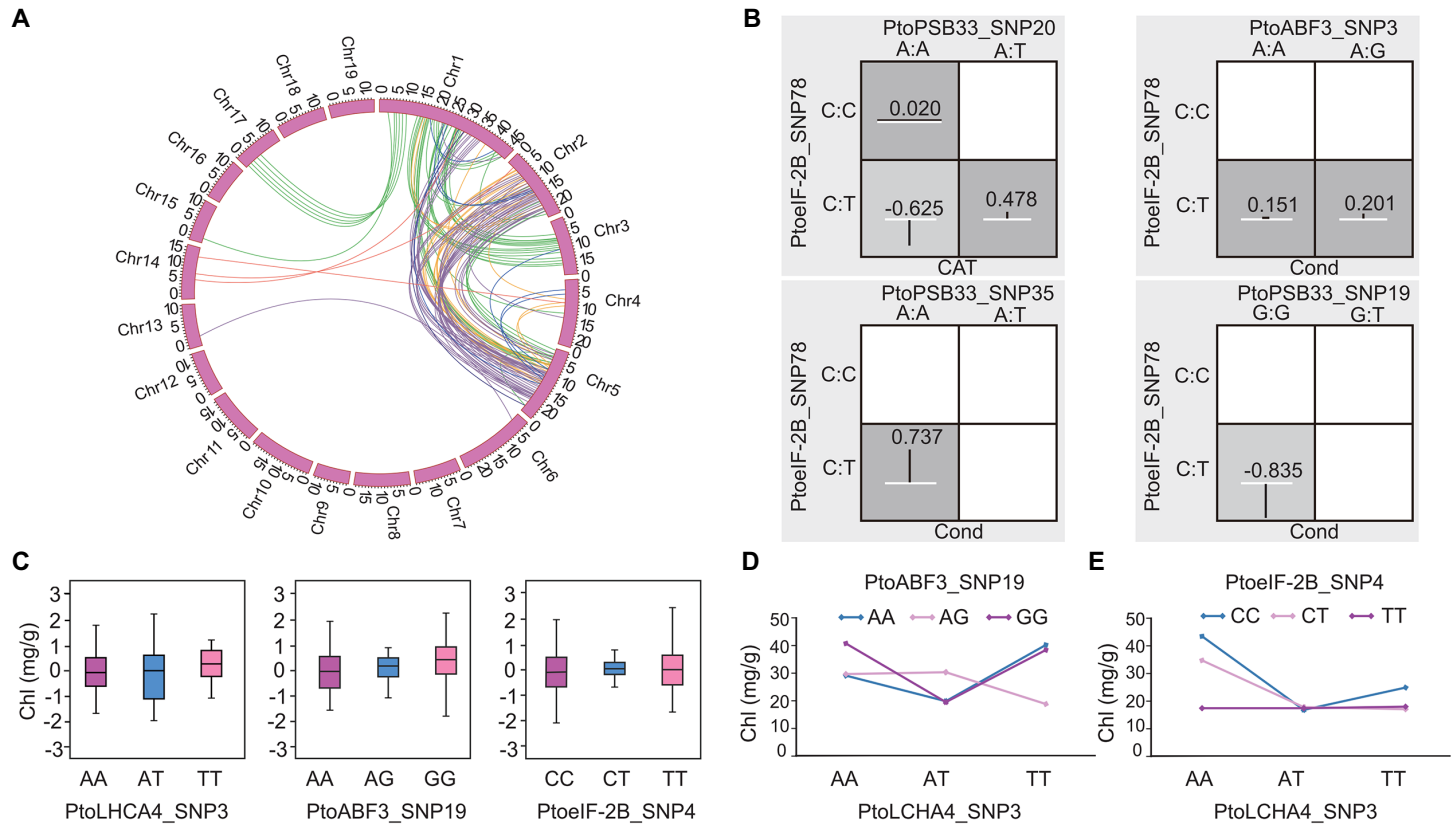
Allelic interactions between significant pairwise SNPs in candidate genes related to the co-expression network of drought stress traits. **(A)** Circos plot showing 104 pairwise interactions for drought stress-related traits (*p* < 0.001). The 19 *P. tomentosa* chromosomes (Chr1-19) are shown in a circle. Interior lines represent the pairwise interactions that underlie six drought stress-related traits; colored lines represent different categories [green, purple, orange, red, dark blue, and light blue indicate relative chlorophyll content (Chl), stomatal conductance (Cond), net photosynthesis (Pn), proline content (PRO), transpiration rate (Trmmol), and catalase activity (CAT), respectively]. **(B)** Epistatic effects of different genotypic combinations for drought stress-related traits. Dark-shaded cells represent high-risk genotype combinations, while light-shaded cells represent low-risk genotype combinations. Values in boxes are individual information gains and positive values along the line indicate positive interactions. The white line in the middle of each box represents the mean phenotypic value of the population, while the vertical line represents the difference between the mean phenotypic value of each genotype combination and the overall mean. The width of the vertical line in the box indicates the number of individuals in this genotype combination. The negative values can be explained as negative interaction/redundancy (i.e., the amount of information shared by the attributes). **(C)** Genotypic effects of chlorophyll content (Chl) causal SNPs. **(D,E)** Epistatic effects for chlorophyll content between PtoLHCA4_SNP3 with PtoABF3_SNP19 and PtoeIF-2B_SNP4.

Epistasis is a critical component of the genetic basis of quantitative traits because it defines the non-additive interactions between variants or genes (Mackay, 2013). To decipher the genetic networks in the response to drought stress, epiSNP was used to assess the epistatic effects of SNP–SNP pairs (Ma et al., 2008). In total, 104 significant pairwise associations (*p <* 0.001) were identified; these associations involved six drought stress-related traits with 95 unique SNPs from 21 genes (Supplementary Table S14). Kempthorne partitioned Fisher’s epistasis effect into four components—additive × additive, additive × dominance, dominance × additive, and dominance × dominance—with the genetic interpretation of allele × allele, allele × genotype, genotype × allele, and genotype × genotype interactions, respectively (Mao et al., 2006). These interactions were partitioned into additive × additive (19 pairs), additive × dominant or dominant × additive (75 pairs), and dominant × dominant (10 pairs; Supplementary Table S14). Additionally, 11 significantly associated genes were repeatedly found to exhibit epistatic effects (104 pairwise), including 10 genetic variants that showed additive/dominant effects. For example, PtoSEC_SNP62 and PtoSEC_SNP75 showed combined additive and dominant effects for Chl. Moreover, they displayed epistatic interactions with PtoeIF-2B_SNP116 on CAT and Cond (Supplementary Table S14). In a total of 43 SNPs showed epistatic interactions with 2-20 SNPs and 11 SNP–SNP pairs were associated with more than one trait. For example, epistatic effects were detected for PtoPSB33_SNP20 (A/T) and PtoeIF-2B_SNP78 (C/T) on CAT and PtoABF3_SNP3 (A/G) with PtoeIF-2B_SNP78 (C/T) for Cond. Additionally, PtoeIF-2B_SNP78 (C/T) interacted with PtoPSB33_SNP35 (T/A) and PtoPSB33_SNP19 (T/G), both of which interacted with Cond (Figures 3A,B). The different allelic interactions showed distinct effects under drought stress. These results suggested that *PtoeIF-2B* (putative translation initiation factor eIF-2B epsilon subunit) had pleiotropic effects on several traits in response to drought stress.

Different genotypic combinations of SNP–SNP pairs had distinct epistatic effects. For instance, Chl varied across different genotypic interactions of PtoABF3_SNP19 (A/G) and PtoLHCA4_SNP3 (A/T); their mean differences in phenotypic values ranged from 18.83 mg/g (AG-TT) to 40.74 mg/g (GG-AA) (Figure 3D). Notably, PtoeIF-2B_SNP4 (C/T) and PtoLHCA4_SNP3 (A/T) also showed epistatic interaction on Chl, but the mean phenotypic values of each genotypic interaction were distinct, ranged from 19.06 mg/g (CC-AT) to 51.27 mg/g (CC-AA) (Figure 3E). The phenotypic values of various genotypic combinations differed from the values of single SNP effects (Figures 3C–E). These epistatic networks of significant drought stress-responsive genetic factors provide alternative effect models for photosynthetic and enzyme activity traits in *P. tomentosa*.

### Genetic regulation of gene expression explains a substantial proportion of the phenotypic variations in response to drought stress of *Populus*

**Figure 4:**
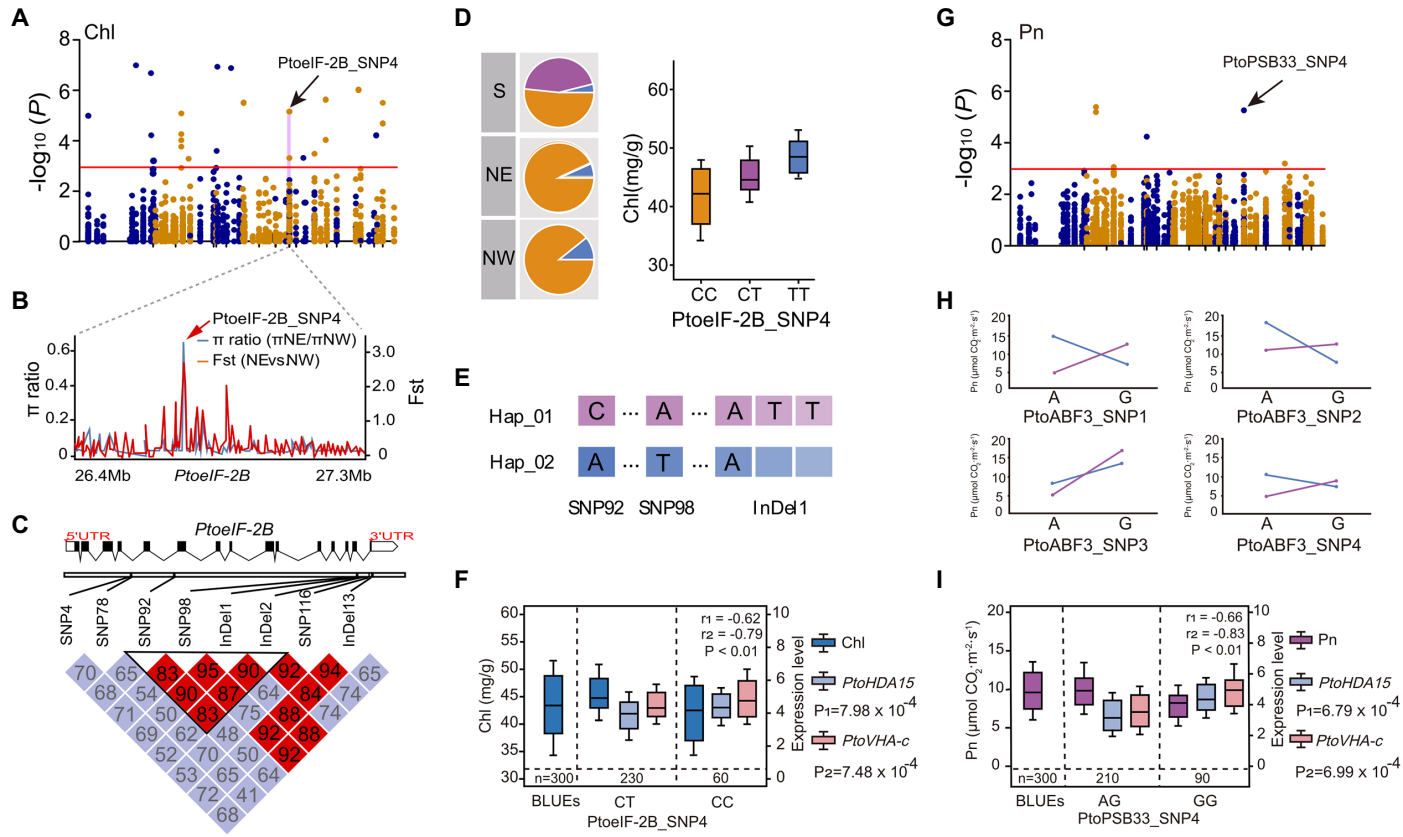
*PtoPSB33* and *PtoeIF-2B* are implicated in the response to drought stress in *P. tomentosa*. **(A)** Manhattan plot for Chl. **(B)** Identification of the selection sweep signature of PtoeIF-2B_SNP4. **(C)** The genome structure and haplotype block of *PtoeIF-2B* gene. **(D)** Genotypic frequencies of significant SNP of *PtoeIF-2B* among the three regions. **(E)** Three significant loci detected by single-gene association analysis, which constituted a conserved haplotype. **(F)** Box plots for Chl (dark-blue), *PtoHDA15* expression (blue) and *PtoVHA-c* expression (pink) plotted as an effect of genotype at PtoeIF-2B_SNP4. **(G)** Manhattan plot for Pn marked with the focal SNP of PtoPSB33_SNP4. **(H)** The epistatic effects of PtoPSB33_SNP4 with four SNPs in *PtoABF3*. **(I)** Box plots for Pn (purple), *PtoHDA15* expression (blue) and *PtoVHA-c* expression (pink) plotted as an effect of genotype at PtoPSB33_SNP4.

To explore the regulatory interactions between allelic variants and expression levels of candidate genes, eQTN mapping was conducted between 5,553 common SNPs (minor allele frequencies >0.05 and missing data <20%) and the expression levels of 75 candidate genes under drought stress. At the threshold of *p <* 0.001 and *q* < 0.05, 319 SNP-gene pairs were identified; thus, 194 unique SNPs in 35 candidate trans-acting factors were associated with the expression levels of 45 candidate genes (Supplementary Table S15). In a total of 52 SNPs were associated with the expression levels of 2–11 genes, suggesting that the expression levels of these candidate genes are under complex genetic regulation. For example, the trans-eQTN *PtoPSB33* (photosystem II protein 33, PtoPSB33_SNP4) was significantly associated with Pn and determined the expression levels of four genes: *PtoPAT23* (protein S-acyl transferases), *PtoPTBP3* (polypyrimidine tract-binding protein), *PtoVHA-c* (vacuolar adenosine triphosphate synthase family protein), and *PtoHDA15* (histone deacetylase). The *PtoHDA15* and *PtoVHA-c* expression levels were negatively correlated with Pn (Figure 4I, Supplementary Tables S12, S14), suggesting that *PtoPSB33* indirectly serves as a master regulator or mediates the leaf physiological response to drought stress.

**Figure 5:**
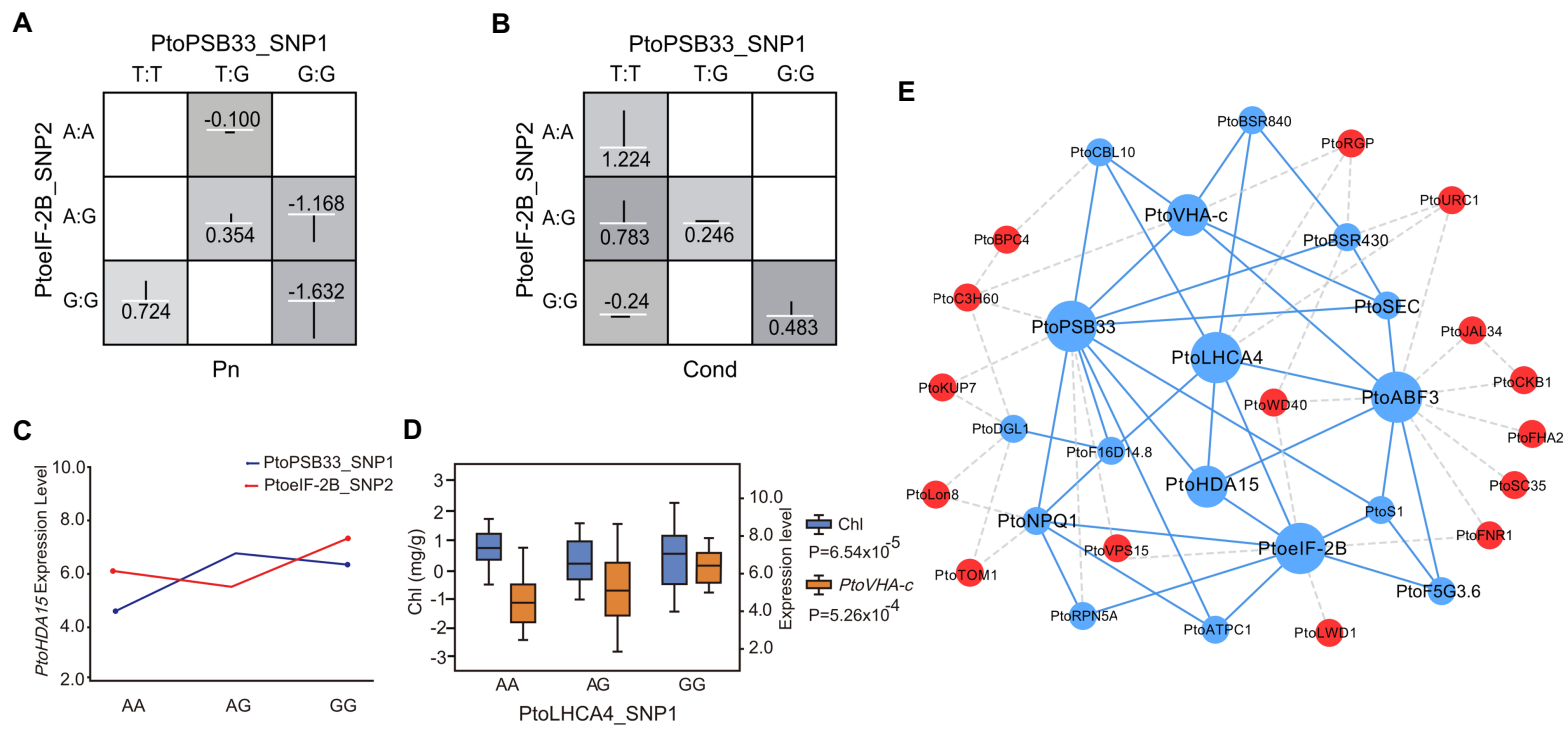
Integration of epistasis and eQTN analyses identifies factors involved in the response to drought stress. **(A)** and **(B)** Photosynthetic-trait epistatic effects of different genotypic combinations. **(C)** Pairwise interactions between PtoPSB33_SNP1 and PtoeIF-2B_SNP2 associate with the expression of *PtoHDA15* with different genotypic combinations at the two loci. **(D)** Box plots for Chl (blue) and *PtoVHA-c* expression (orange) plotted as an effect of genotype at PtoLHCA4_SNP1. **(E)** Proposed network of interactions among genetic factors. Putative regulatory network of candidate genes constructed by association mapping and co-expression analysis. Blue circles represent genes with epistatic interactions while red circles represent association mapping-verified regulators. Dotted lines indicate potential interactions, including co-expression interactions; solid lines indicate interactions verified by association mapping and co-expression analysis.

In addition, a total of 17 SNPs detected by single SNP-based association studies (Supplementary Table S13), and 29 SNPs detected by the epistasis model overlapped with SNPs identified by eQTN analysis (Supplementary Table S14). For example, PtoPSB33_SNP1 and PtoeIF-2B_SNP2 formed an epistatic interaction with Pn and Cond (Figures 5A,B). Moreover, both were associated with the expression level of *PtoHDA15* (Figure 5C), which could contribute to the photosynthetic traits. Therefore, epistatic effects in the genetic architecture of complex quantitative traits have been overlooked. PtoLHCA4_SNP1 was associated with Chl and the expression level of *PtoVHA-c* (Figure 5D), suggesting that *PtoLHCA4* probably contributed to the phenotypic variation. Alternatively, these factors might also affect photosynthetic traits by regulating the expression levels of other genes. Therefore, the combination of association mapping and eQTN mapping enabled the evaluation of the genetic interactions and regulatory networks of the leaf physiological response to drought stress.

### Determination of the four conserved genes that respond to drought stress in *Populus*

By combining the results of association mapping, eQTN, and co-expression analyses, we evaluated the putative functions of the genes involved in drought stress. We detected the candidate gene *PtoeIF-2B* in two major variants (PtoeIF-2B_SNP4 and PtoeIF-2B_InDel1). PtoeIF-2B_SNP4 (C/T) was a significant SNP for Chl (*p* = 7.20 × 10^−5^, *q* < 0.05, *R*^2^ = 21.94%; Figures 4A,B). The frequency of allele (TT) from PtoeIF-2B_SNP4 for higher Chl increased from the S (5.6%) geographical region to the NE (14.8%) and NW (20.6%) geographical regions, suggesting that *PtoeIF-2B* is subjected to adaptive selection in response to the local environment (Figure 4D; Supplementary Table S16). To assess the functional roles of *PtoeIF-2B*, we conducted a haplotype analysis and the results revealed PtoeIF-2B_InDel1 and two other SNPs (PtoeIF-2B_SNP92 and PtoeIF-2B_SNP98) in *PtoeIF-2B* that constitute the haplotype were significantly associated with drought stress-related traits. Hap_01PtoeIF-2B (C-A-ATT) was identified as the superior haplotype associated with Pn; the mean value for Hap_01PtoeIF-2B that was 12.0% higher than the mean value for Hap_02PtoeIF-2B (*p* < 0.05; Figures 4C–E; Supplementary Table S17). Furthermore, PtoeIF-2B_SNP4 (C/T) was a trans-eQTN for the expression levels of *PtoHDA15* and *PtoVHA-c* which were negatively correlated with Chl (*r*_1_ = −0.62 and *r*_2_ = −0.79, respectively, *p* < 0.01; Figure 4F). Notably, we detected seven strong eQTN signals that were associated with the expression levels of *PtoHDA15* and *PtoVHA-c*. Of them, three eQTNs (PtoeIF-2B_SNP4, PtoABF3_SNP19, and PtoLHCA4_SNP3) showed significant epistatic interactions with Chl; different combinations of these three SNPs had distinct contributions to the expression levels of *PtoHDA15* and *PtoVHA-c* (Figure 3D), implying that the three candidate genes might indirectly affect Chl. Additionally, the expression levels of *PtoHDA15* and *PtoVHA-c* were associated with PtoABF3_SNP1 and PtoLHCA4_SNP4 which displayed potential epistatic interactions for Pn (Supplementary Table S14). These results suggest that *PtoeIF-2B*, *PtoABF3*, and *PtoLHCA4* may associate with the expression levels of *PtoHDA15* and *PtoVHA-c*, which, in turn, may affect Pn by regulating Chl (Figure 5E; Supplementary Table S14).

Association mapping showed that PtoPSB33_SNP4 (G/A) was significantly associated with Pn (*p* = 7.01 × 10^−5^, *q <* 0.05, *R*^2^ = 12.15%) and epistatic interact with four SNPs (PtoABF3_SNP1, PtoABF3_SNP2, PtoABF3_SNP3, and PtoABF3_SNP4) in *PtoABF3* for Pn (Figures 4G,H). Moreover, *PtoPSB33* and *PtoABF3* both had eQTNs associated with the expression levels of *PtoHDA15* and *PtoVHA-c*, which were negatively correlated with Pn variation (*r*_1_ = −0.66, *r*_2_ = −0.83, respectively, *p* < 0.01) (Figure 4I). In total, four candidate genes (*PtoeIF-2B*, *PtoABF3*, *PtoPSB33*, and *PtoLHCA4*) were identified by both association mapping and co-expression network analysis; they were considered hub genes for regulating the drought stress response in poplar. A phylogenetic tree based on the protein sequences of the four genes showed that all clustered in the same group in the five poplar species, indicating that they were highly conserved in poplar (sequence similarities 95.93%–99.42%; Supplementary Figure S5). In addition, the expression patterns of these four potential hub genes were similar in the five poplar species (Supplementary Figure S6). Finally, we identified four hub candidate genes (*PtoeIF-2B*, *PtoABF3*, *PtoPSB33*, and *PtoLHCA4*) that formed a conserved network with *PtoHDA15* and *PtoVHA-c* during the response to drought stress (Supplementary Figure S7). *PtoeIF-2B* is a potential regulator associated with the expression levels of *PtoHDA15* and *PtoVHA-c* (Figure 4F). Moreover, *PtoeIF-2B* showed significant epistatic interactions with *PtoABF3* and *PtoLHCA4* (Figures 3B,E), different genotypic combinations had distinct contributions to the expression levels of *PtoHDA15* and *PtoVHA-c*. *PtoPSB33* and *PtoABF3* formed epistatic interaction networks for Pn (Figures 4G,H); they jointly effected the expression levels of *PtoHDA15* and *PtoVHA-c*. Our results suggested that these hub genes have conserved genetic effects in *Populus* on transcription role and phenotypic variations.

## Discussion

Drought stress is one of the most drastic abiotic stresses in plants, and regulatory factors that mediate the response to drought stress have been identified by reverse genetics (Zhou et al., 2007; Bang et al., 2018). Here, we used forward genetics to identify four important genes in the response to drought stress in *Populus*; our work provides an important theoretical foundation for the genetic improvement of drought stress tolerance in forest trees. The drought stress response is a complex multi-gene process. Thus, we used co-expression analysis, association genetics, and eQTN mapping to explore the genetic regulatory network of the drought stress response in *Populus*. We identified a conserved network and four key genes that are putatively involved in the drought response in *Populus*. This strategy enabled the identification of modules of co-expressed genes across multiple spatial, temporal, and environmental conditions, thereby providing insights into the co-expression network and candidate genes potentially responsible for the plant drought stress response.

### Physiological and transcriptional regulation responses of *Populus* to drought stress

The drought stress response involves hydraulic signals, reactive antioxidants, osmotic regulation, and phytohormone movement processes (Attipalli et al., 2004; Ahmad et al., 2010). Compared with annual crops, perennial forest trees are exposed to long-term, complex external environmental conditions; they have evolved adaptive traits to manage drought stress (Lu et al., 2021). In this study, Pn, Cond, and Trmmol decreased under drought conditions (Supplementary Table S1). In contrast, we found that plant leaves had reduced Chl under drought stress, which might explain their lower rate of photosynthesis (Guo et al., 2018). The correlations among the six drought stress-related traits suggest that these traits jointly response to drought stress. In addition, the phenotypes of six drought stress-related traits displayed considerable variation, with the coefficient of variation values that ranged from 0.15 to 7.59 and *H*^2^ values that ranged from 0.29 to 0.87 (Supplementary Table S1). Collectively, the above findings demonstrated that the six drought stress-related traits were suitable for investigating the genetic control of poplar drought tolerance by association mapping.

Transcriptionally co-regulated and functionally related genes have been identified by co-expression analysis. The transcriptomic profile of the genus *Populus* under drought stress has been investigated (Street et al., 2006; Viger et al., 2016; Garcia et al., 2019). We evaluated the transcriptome profiles of five *Populus* species under multiple spatial, temporal, and drought stress conditions to identify drought stress response genes that were conserved during the evolution of angiosperms. Our co-expression network analysis revealed 75 candidate genes that were implicated in the response to drought stress, with edge weight ≥ 0.5 and node connectivity ≥10 in the network (Supplementary Table S9). Some of these genes were reported to participate in the drought stress response, consistent with the observation that drought stress involves a complex regulatory network (Georgii et al., 2019). For example, *ABF3* plays an important role in the regulation of the drought response by interacting with the ABA-independent proteins DREB2A, DREB1A, and DREB2C in the *Arabidopsis* (Liu et al., 2018). *PtoVHA-c* confers stress tolerance by enhancing superoxide dismutase and peroxidase activities under drought stress (Feng et al., 2015). In addition, many novel high-degree hub gene signatures were identified in our analysis. For example, *RPN5A* is a 26S proteasome subunit, which degrades a wide range of intracellular proteins (Book et al., 2009). These findings underscore the ability of co-expression analysis to identify genes implicated in the responses to abiotic stresses.

### Multi*-*omics analysis as a high*-*confidence strategy to assess drought stress

The drought stress response in trees involves multiple interconnected molecular pathways, which modulate various cellular functions (Gupta et al., 2020). Unlike most crops, trees form large continuous natural populations in highly heterogeneous environments that harbor significant genetic diversity, thus promoting phenotypic response to environmental (Di Filippo et al., 2015). Therefore, the construction of a drought stress systematic regulatory network and identification of potential regulatory genes would provide insights into the evolutionary background of perennial trees and promote plant breeding on the basis of specific regional climate (Lu et al., 2021). We constructed three co-expression network modules, and identified 75 hub genes based on edge weight and node connectivity; our results enabled the identification of a putative master regulator of the drought stress response (Supplementary Table S9). General regulator network based on 75 hub genes was constructed by systematic integration of association mapping analysis in drought stress. The most likely candidate genes in the drought stress response were *PtoeIF-2B*, *PtoPSB33*, *PtoABF3*, and *PtoLHCA4*. This integrative strategy has facilitated the functional interpretation of complex trait-associated signals in other studies and enables the identification of target traits and functionally associate genes (Nica et al., 2010). Furthermore, epistasis analysis allows the identification of the functional allele pairs that contribute to drought stress traits (Xiao et al., 2019). *PtoeIF-2B*, *PtoABF3*, and *PtoLHCA4* showed significant epistatic interactions affecting Chl. Different genotypic combinations of *PtoeIF-2B*, *PtoABF3* had distinct contributions to the expression levels of *PtoHDA15* and *PtoVHA-c* expression (Figure 4; Supplementary Table S14). Indeed, *eIF-2B* and *Lhca4* were down-regulated under drought stress in a drought sensitive wheat cultivar (Abbasi et al., 2021). These findings improve our understanding of the role of epistasis in drought stress adaptation in trees (Du et al., 2019).

The analysis of naturally occurring allelic variance and allele frequency in different climatic regions provides insights into adaptive evolution (Mitchell-Olds and Schmitt, 2006). Here, we found PtoeIF-2B_SNP4 was annotated as the important drought stress-responsive gene *PtoeIF-2B*, located in a selective sweep region; the frequency of allele PtoeIF-2B_SNP4 (increased chlorophyll content) increased from S, to NE, to NW. This suggested that genetic loci related to chlorophyll content might be associated with drought stress adaptation in *Populus* (Supplementary Table S16). Therefore, these selected loci showed significant regional differentiation, highlighting the potential roles in the response to drought stress (Kurasch et al., 2017). Haplotype data from population samples contain information regarding the history of allelic associations, which may aid in forest tree conservation (Leitwein et al., 2019). In this study, the haplotype frequency was higher in individuals in the NW than in the S or NE, implying that the lower annual rainfall led to selection for Hap_01. Combining co-expression analysis, association analysis, and eQTN mapping enabled analysis of quantitative traits in complex regulatory networks. Using this strategy, we constructed a genetic network of genes in trees under drought stress, which will promote forest tree genetic improvement programs and provide diagnostic tools for the conservation and management of natural populations (Neale and Kremer, 2011).

### Functional interpretation of four conserved candidate genes associated with drought stress

Based on a multi-omics strategy, *PtoeIF-2B*, *PtoPSB33*, *PtoABF3*, and *PtoLHCA4* were identified as the four candidate genes response for drought stress in *Populus.* Notably, *PtoPSB33*, *PtoeIF-2B*, and *PtoABF3* showed epistatic interactions occur between allelic variation at different loci, which in turn can have an effect on the traits (Figures 3B, 4H). *PtoPSB33*, *PtoeIF-2B* affected the expression levels of *PtoHDA15* and *PtoVHA-c*, which were negatively correlated with Chl and Pn (Figures 4F,I). Moreover, different genotypic combinations of *PtoLHCA4* had distinct contributions to the expression levels of *PtoVHA-c* (Figure 5D). Therefore, *PtoeIF-2B*, *PtoPSB33*, *PtoABF3*, and *PtoLHCA4* jointly affected the expression levels of *PtoHDA15* and *PtoVHA-c*; they also affected Pn by regulating the Chl content under drought stress, suggesting that these hub genes have conserved genetic effects in five poplar species. Future studies should investigate the allelic coordination between drought stress and physiological functions. *PtoHDA15* and *PtoVHA-c* have important roles in the plant stress response and tolerance (Nakashima and Yamaguchi-Shinozaki, 2013; Kato et al., 2017). Liu et al. (2013) reported that *AtHDA15*, a homolog of *PtoHDA15* in *Arabidopsis thaliana*, acted as a transcriptional repressor and negatively regulated levels of genes linked to chlorophyll biosynthesis and photosynthesis. Our genetic analysis indicated that *PtoeIF-2B*, *PtoPSB33*, *PtoABF3*, and *PtoLHCA4* were key genes in the genetic association network of the response to drought stress in poplar (Figure 5E). We propose the following mechanisms for this association. *PtoeIF-2B*, *PtoABF3*, *PtoLHCA4*, and *PtoPSB33* presumably constitute the initial defense response, which also induces the expression of downstream genes essential for adaptation to environmental stress. In addition, these four candidate genes exhibited significant differentiation during the evolution of dicots and monocots, and they were highly conserved in poplar (Supplementary Figure S5). It is unclear that four candidate genes ancestral by purifying selection, or independent positive selection in different lineages. However, this conservation enables the mining of drought stress-related genes in plant species. The drought stress-related genes in *Populus* identified in our study can serve as a useful resource for other species.

In summary, co-expression analysis, association genetics, and eQTN mapping enable dissection of the complex genetic networks of quantitative traits, such as drought tolerance. This integration strategy is a considerable advancement from the approach to facilitate an integrated conservation genomics approach for assessment of the effects of genetics and environment on adaptive traits (Ouborg et al., 2010). Our method allowed exploration of the association relationships of drought stress genes and provided a basis for understanding the complex genetic regulation involved. However, because the mechanisms that underlie the interactions between epistasis and eQTNs are unclear, functional analyses are required (Ingvarsson and Street, 2010). Continued use of genome-editing techniques in *Populus* will decipher the functions of candidate genes (e.g., *PtoeIF-2B*, *PtoABF3*, *PtoLHCA4*, and *PtoPSB33*) involved in drought stress in *Populus* (Zhou et al., 2015). Further analysis of single nucleotide substitutions will provide insights into the mechanism of allelic interactions in poplar.

## Data availability statement

The original contributions presented in the study are publicly available. This data can be found at: https://ngdc.cncb.ac.cn/, CRA005557.

## Author contributions

DZ designed the experiments, obtained the funding, and is responsible for this article. FS, QD, MQ, LX, and WL performed the experiments. JZ, SQ, FY, DW, and PL collected and analyzed the data. FS wrote the manuscript. YE-K revised the manuscript and provided valuable suggestions concerning the manuscript. All authors contributed to the article and approved the submitted version.

## Funding

The present study was supported by The Major Science and Technology Projects of Inner Mongolia Autonomous Region (2021ZD0008), the Project of the National Natural Science Foundation of China (nos. 31872671 and 32170370), and the 111 Project (no. B20050).

## Conflict of interest

The authors declare that the research was conducted in the absence of any commercial or financial relationships that could be construed as a potential conflict of interest.

## Publisher’s note

All claims expressed in this article are solely those of the authors and do not necessarily represent those of their affiliated organizations, or those of the publisher, the editors and the reviewers. Any product that may be evaluated in this article, or claim that may be made by its manufacturer, is not guaranteed or endorsed by the publisher.

## Supplementary material

The Supplementary Material for this article can be found online at: https://www.frontiersin.org/articles/10.3389/fpls.2022.829888/full#supplementary-material

## Footnotes

^1^ https://www.bioconductor.org/

^2^ http://systemsbiology.cau.edu.cn/agriGOv2/index.php

^3^ https://www.kegg.jp/

^4^ http://www.ncbi.nlm.nih.gov/
