# Supplementary material for: Transcriptome and association mapping revealed functional genes respond to drought stress in Populus
Source: Front Plant Sci. 2022 Jul 29;13:829888. doi: 10.3389/fpls.2022.829888 (PMC9372527; doi:10.3389/fpls.2022.829888)
Supplement: Supplementary file 1 [file Data_Sheet_1.zip › Supplementary Figures.docx]

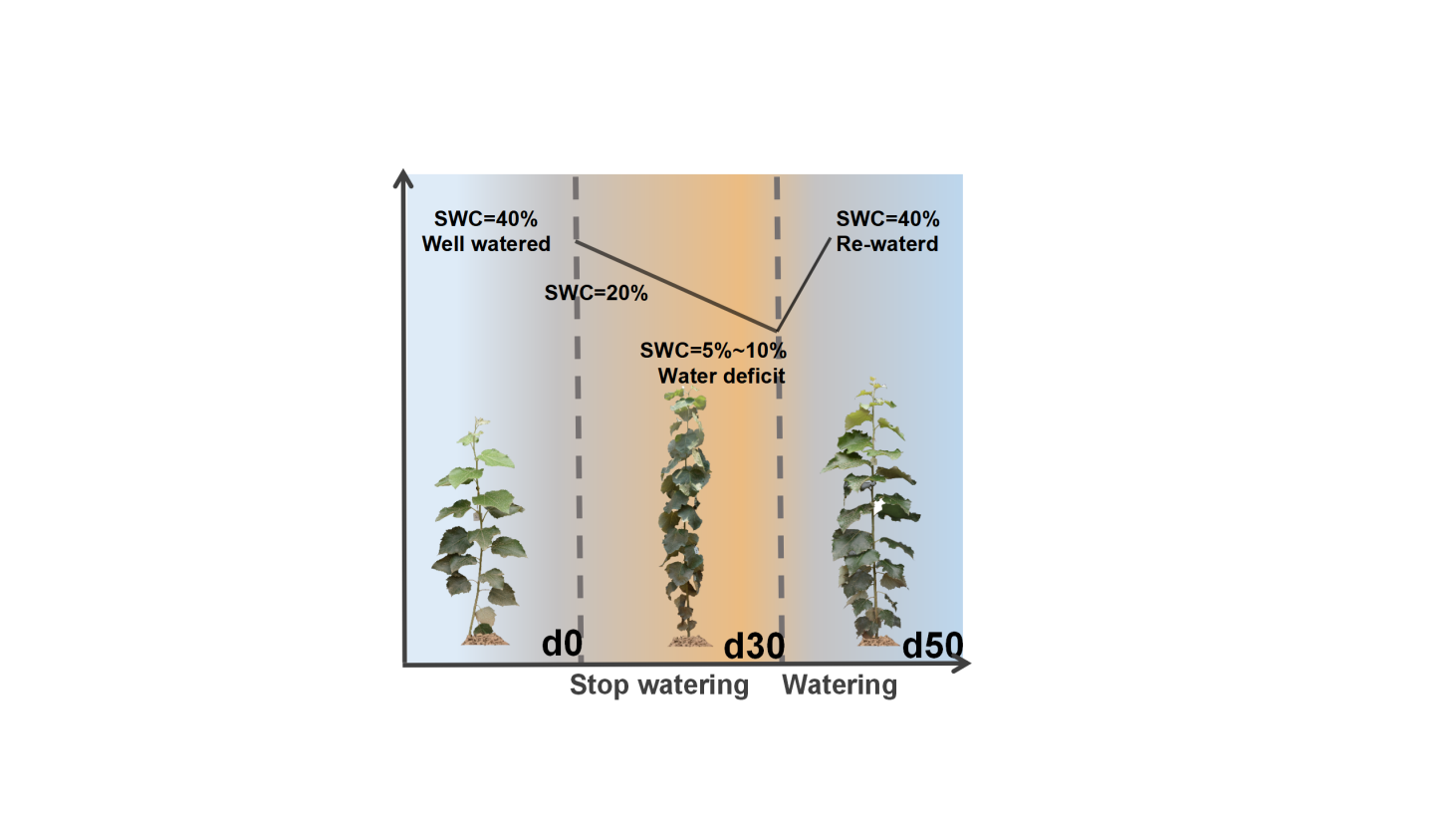


**Figure S1.** Schematic diagram of drought stress process (1) 20 d well-watered (WW); 2) 30 d water deficit (WD); and 3) re-watering (RW) for 20 d three times per week. The black broken line indicates changes in the volumetric soil water content (SWC).


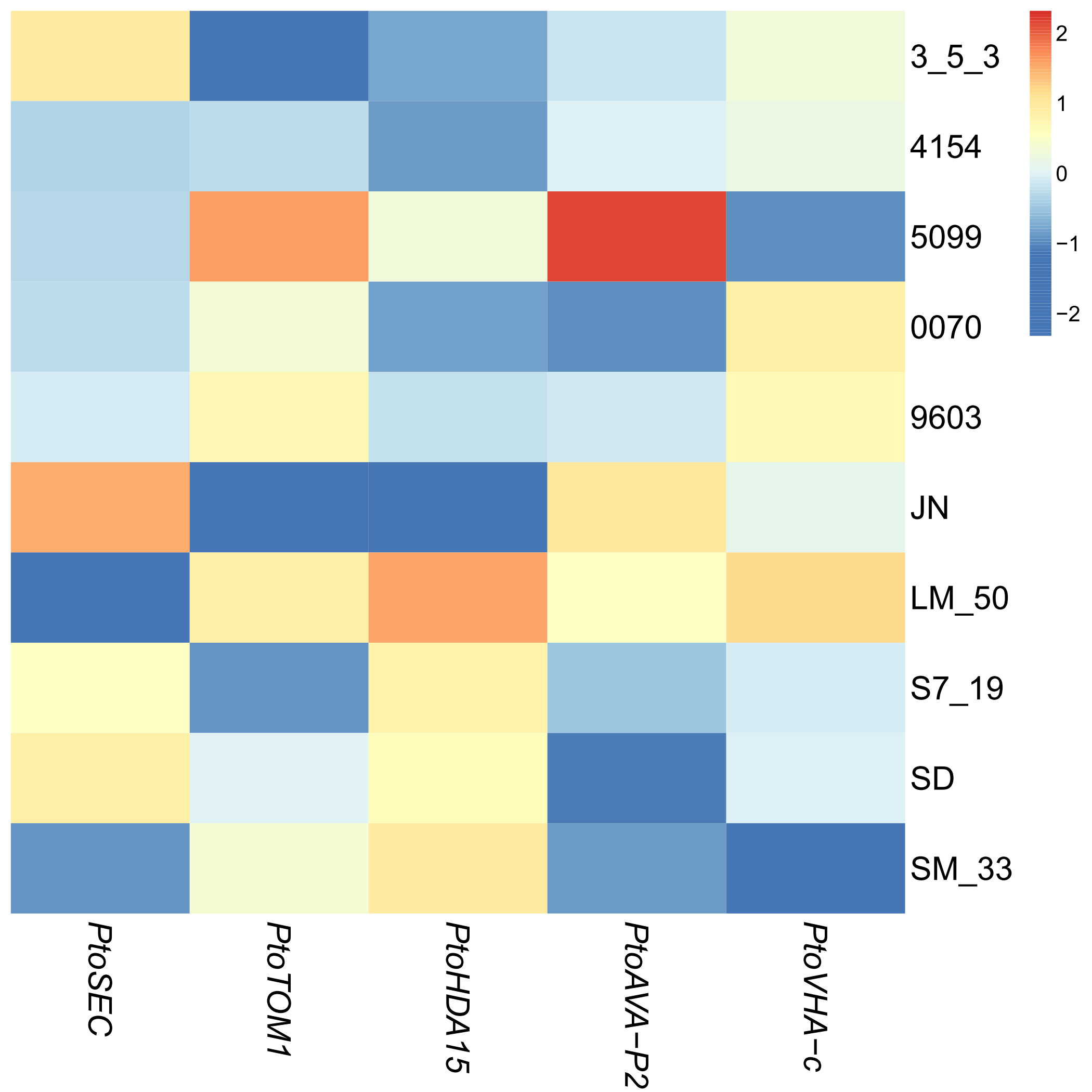

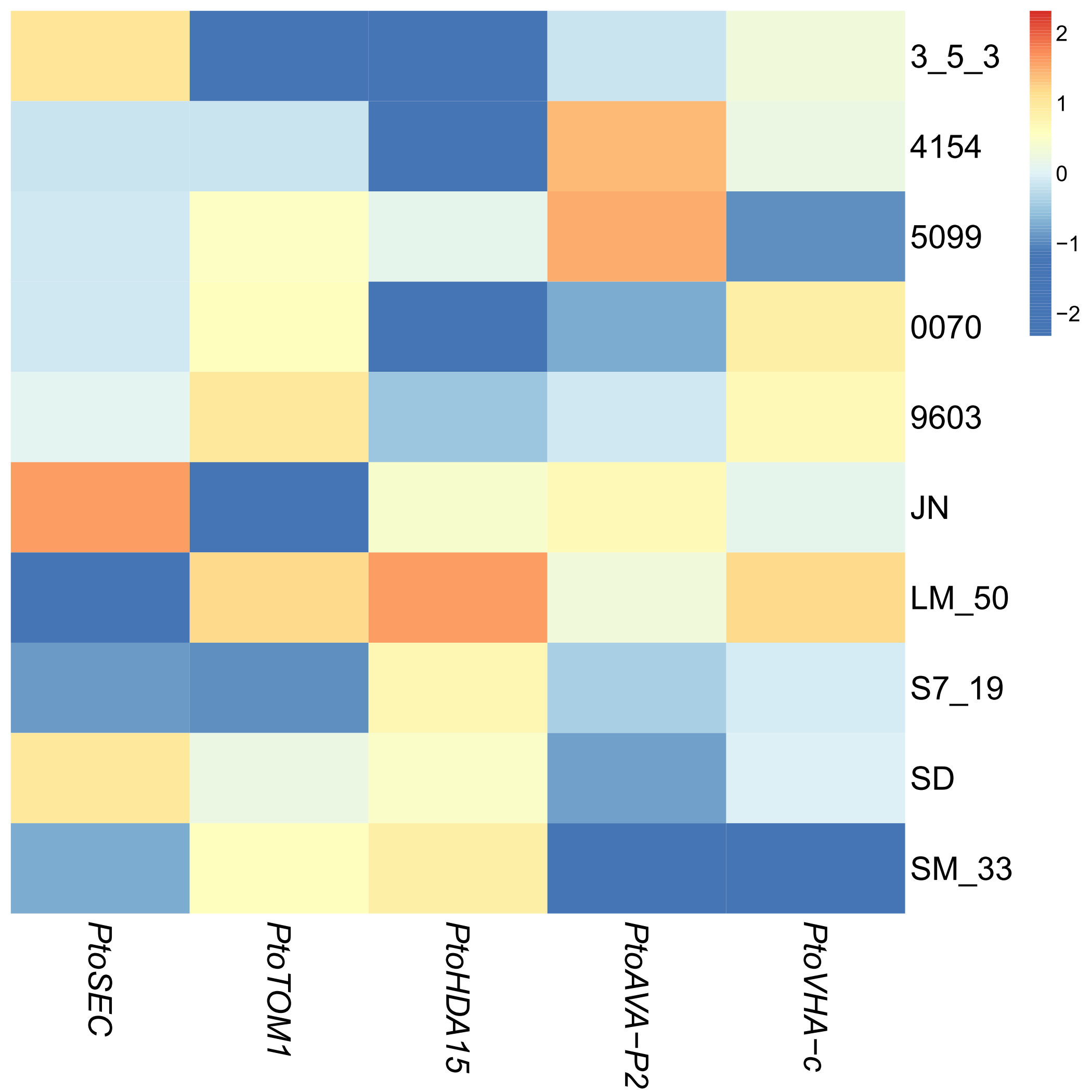


**Figure S2** Transcript abundances of five genes according to RNA-seq (left) and RT-qPCR (right). The codes (e.g. 3_5_3) on the right of the figure means the [serial](javascript:;) [number](javascript:;) of the ten *P. tomentosa.* Redder color blocks indicate higher expression levels, and bluer color blocks indicate lower expression levels.





**Figure S3** Bubble diagram of GO enrichment results for the blue (A), turquoise (B), and brown (C) modules. The *x*-axis represents gene ratio, and the *y*-axis represents GO terms. The size of each circle indicates gene count. The color of circles represents different -log_10_(P-values).


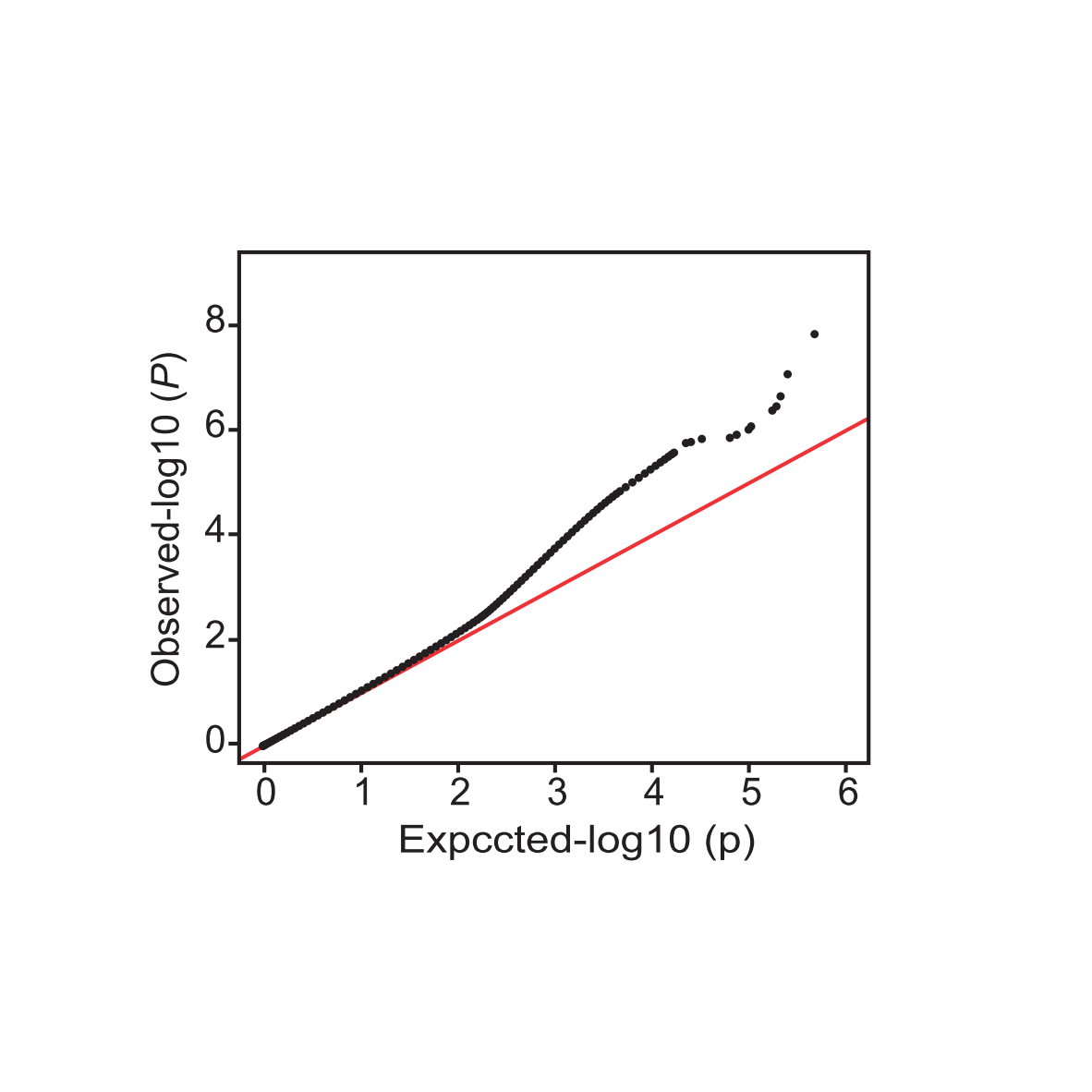

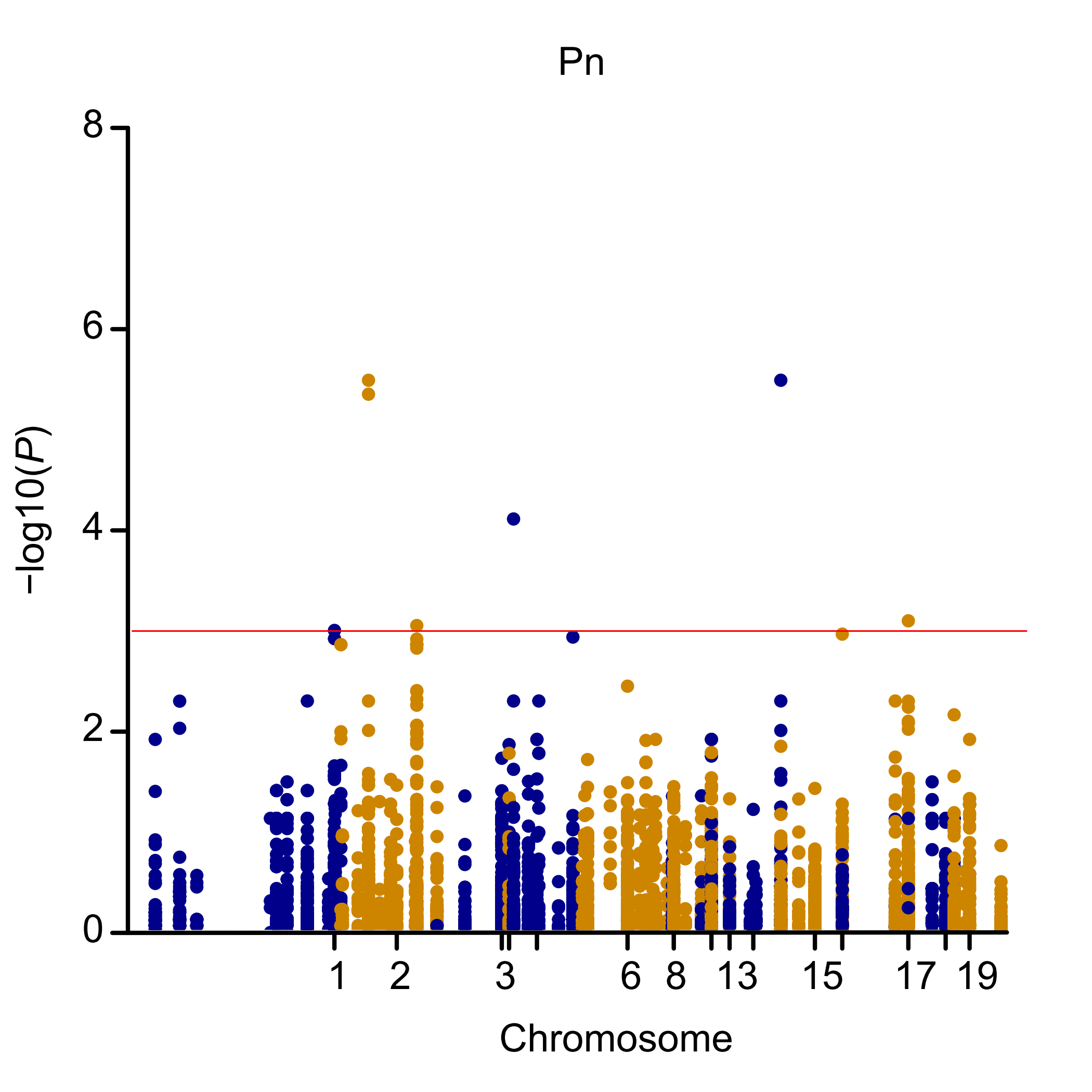

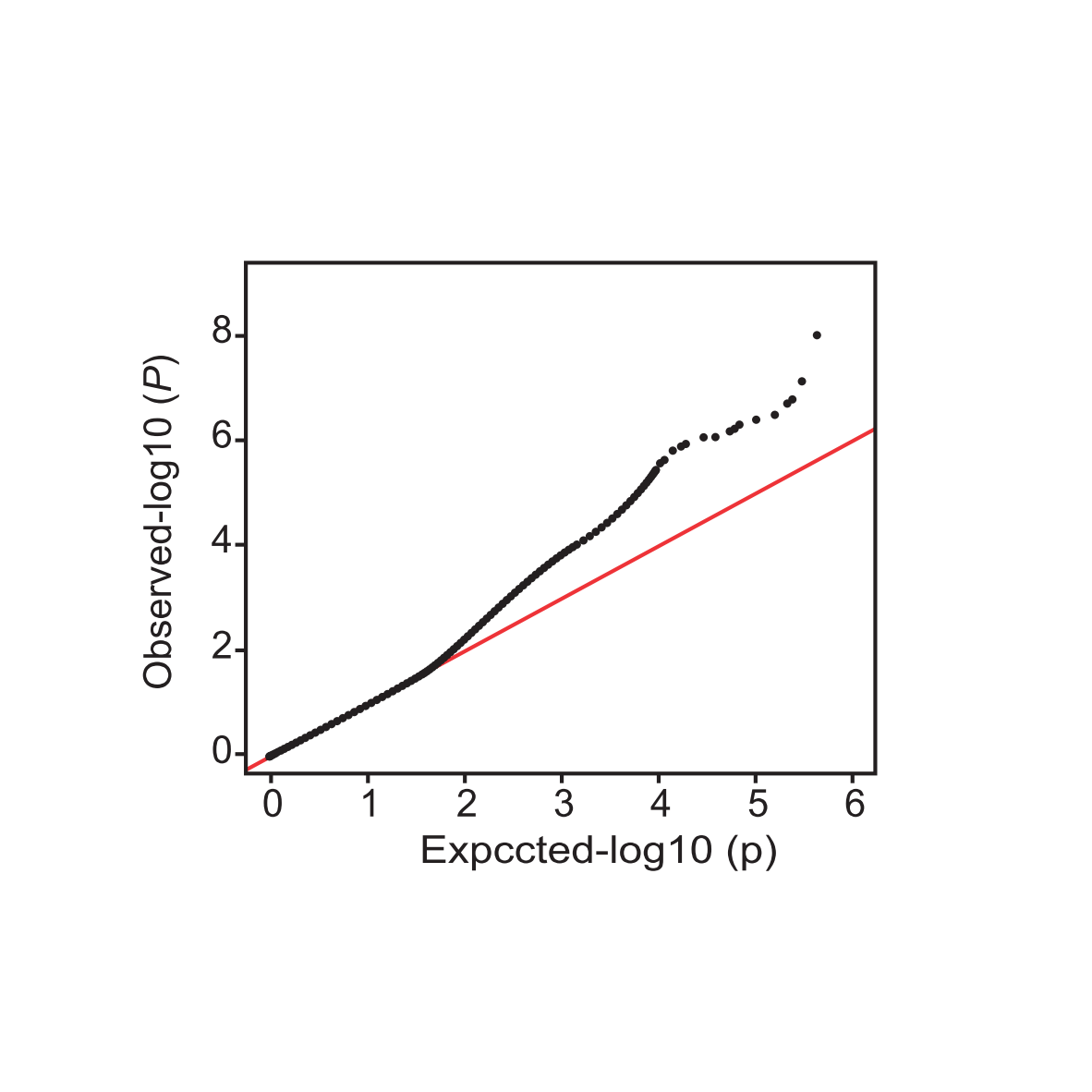

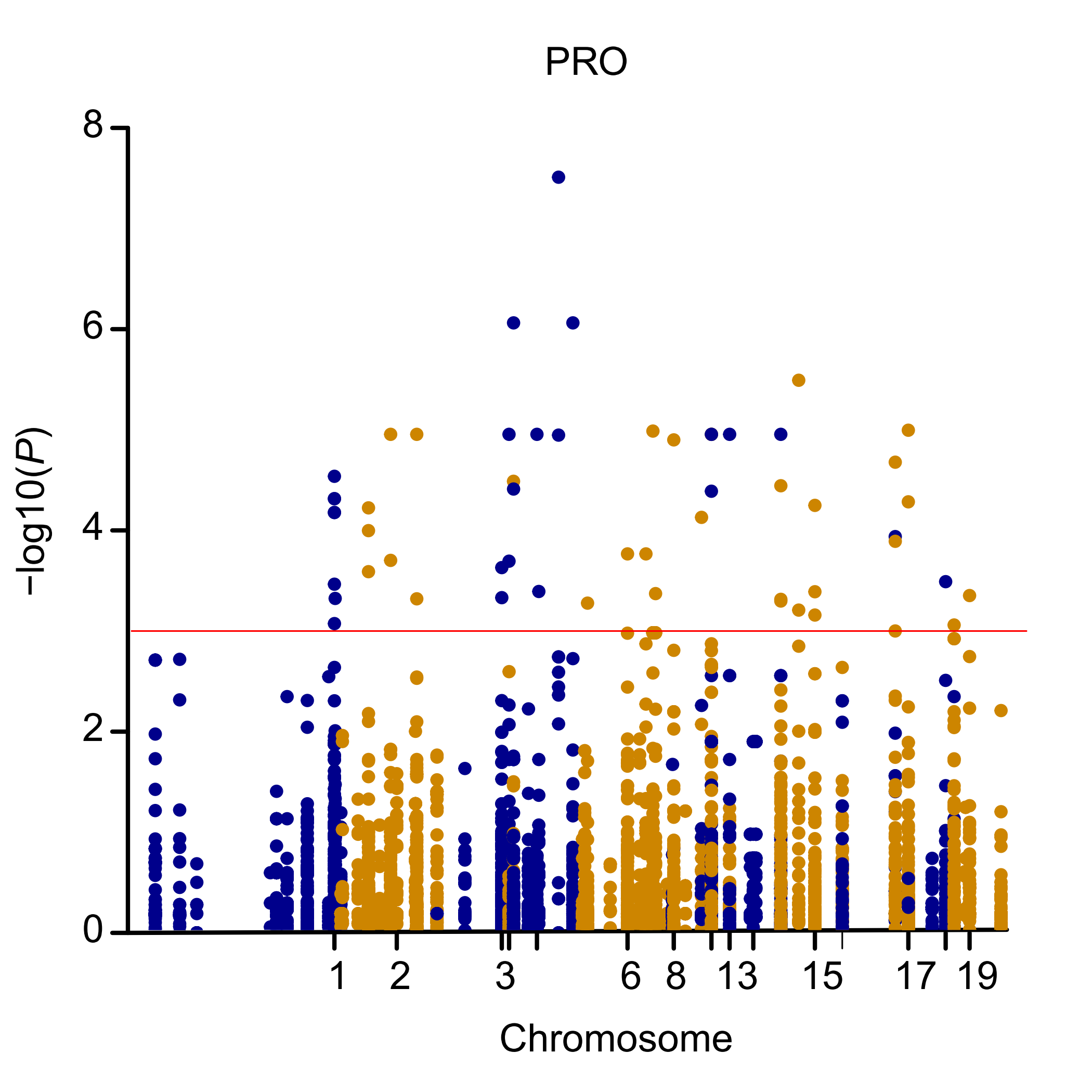

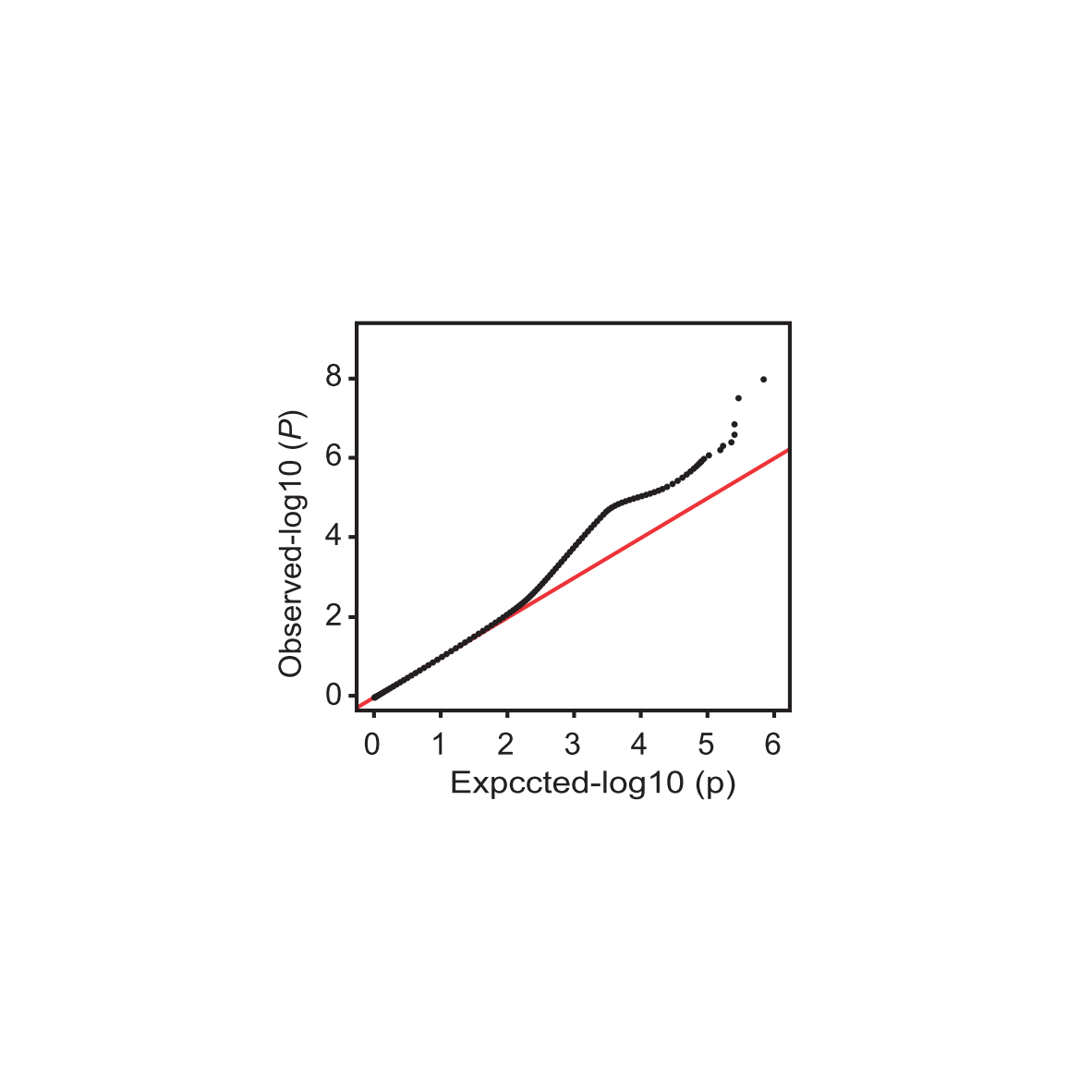

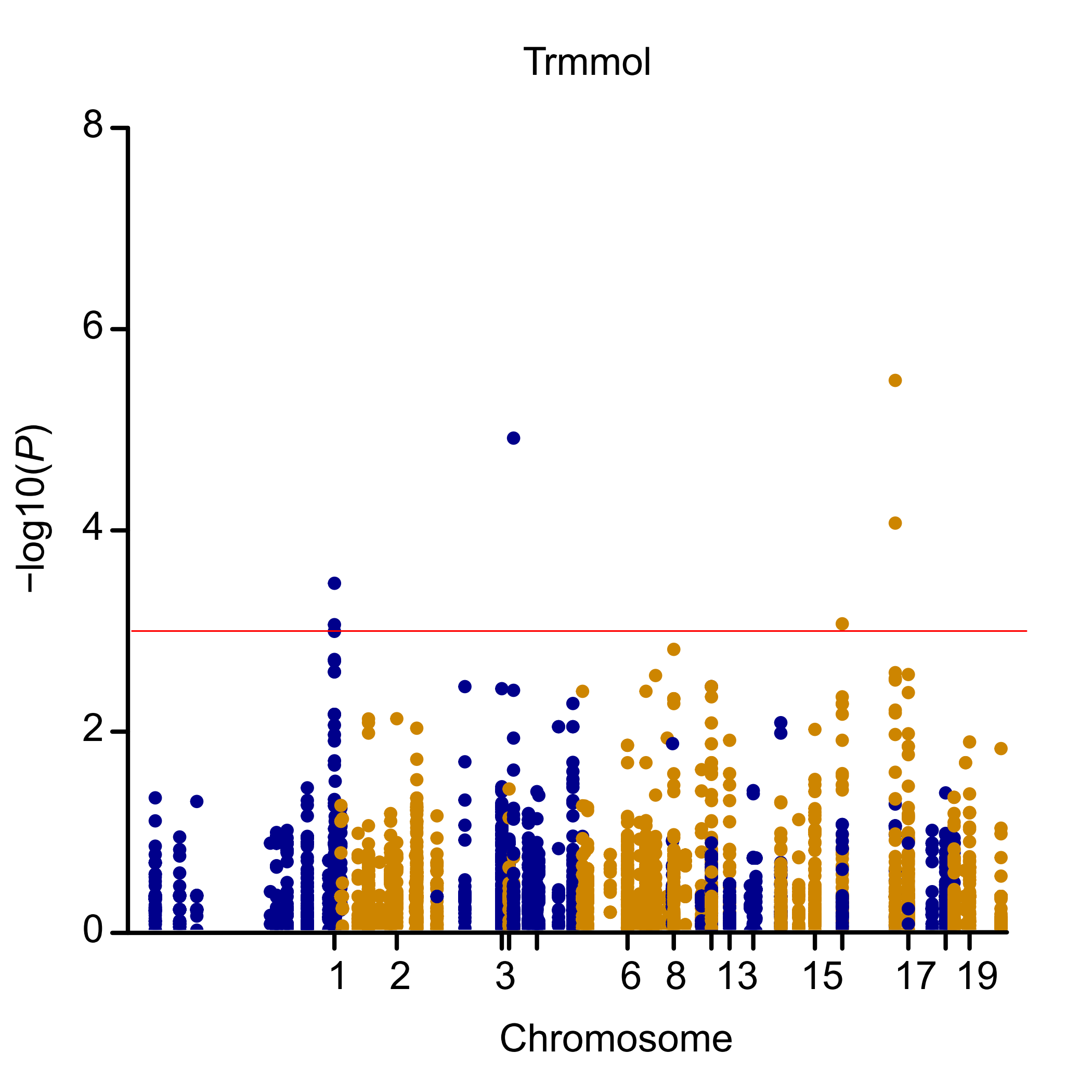

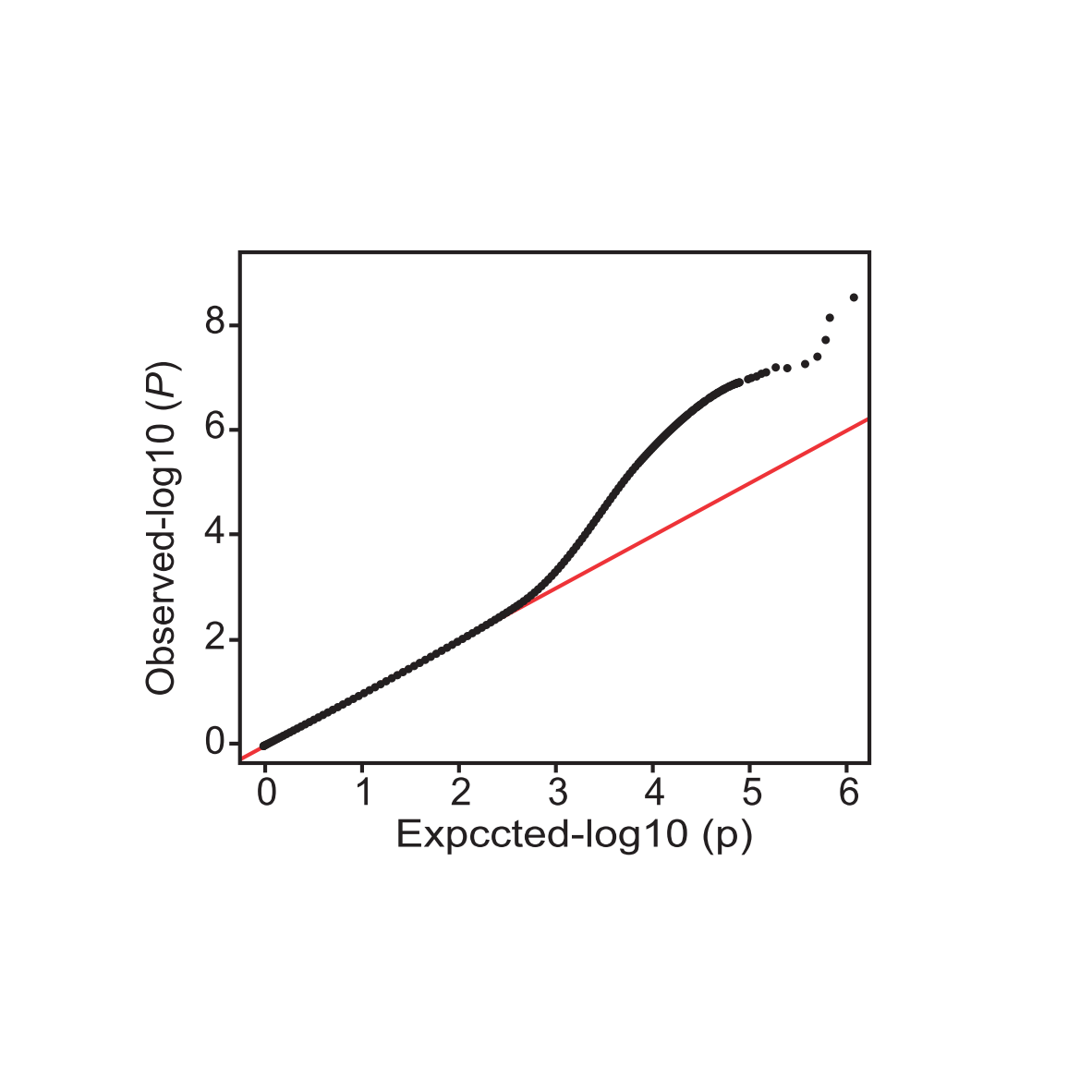

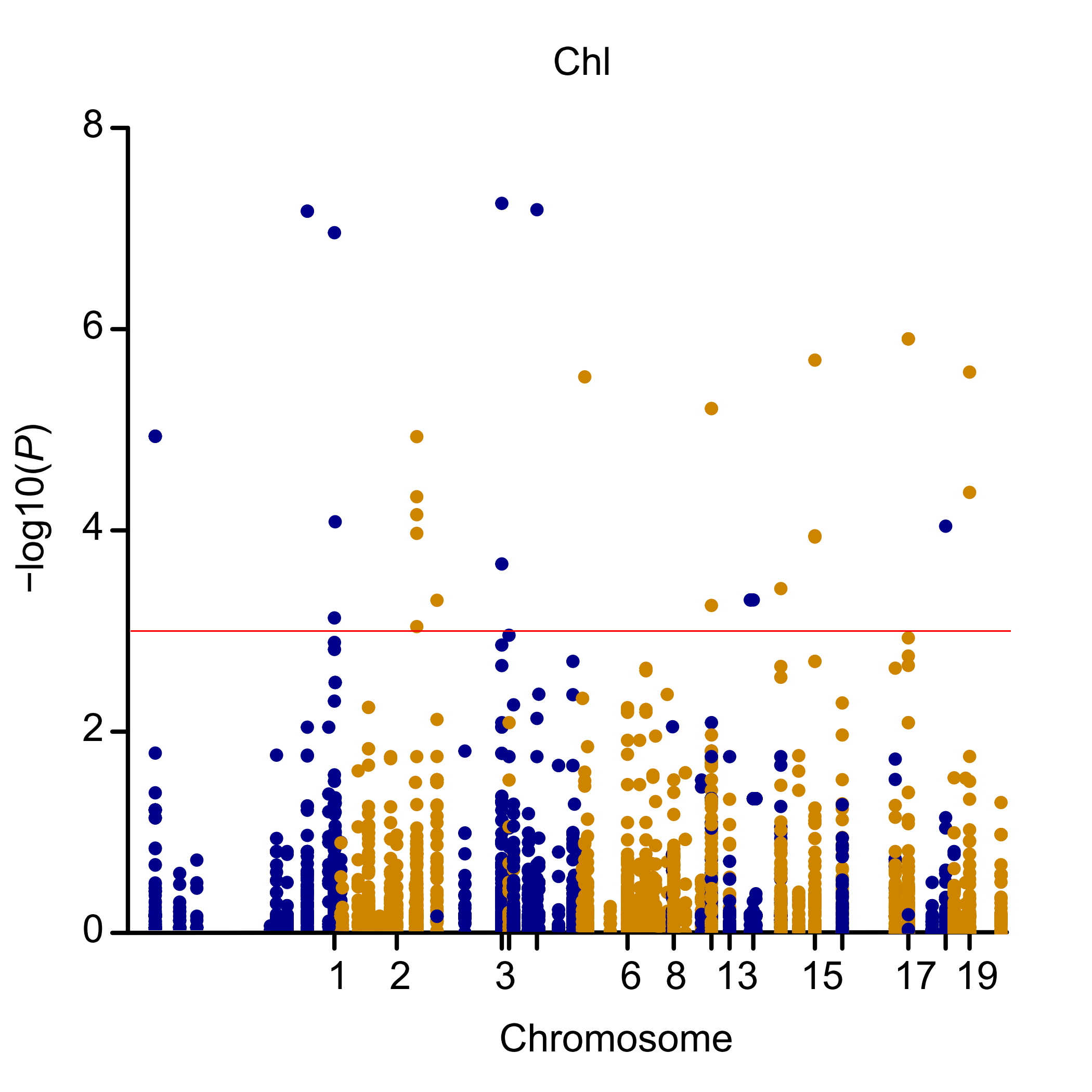

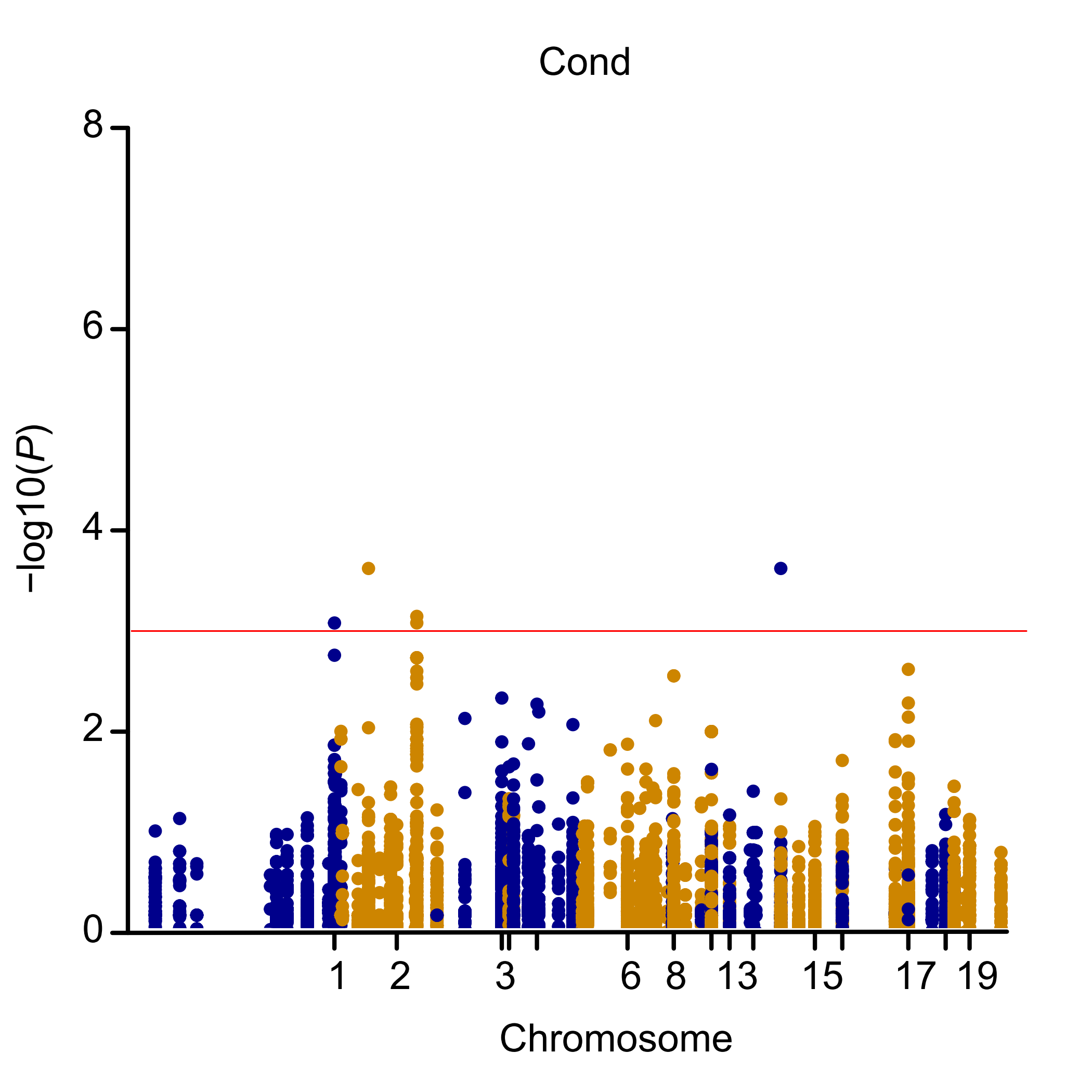

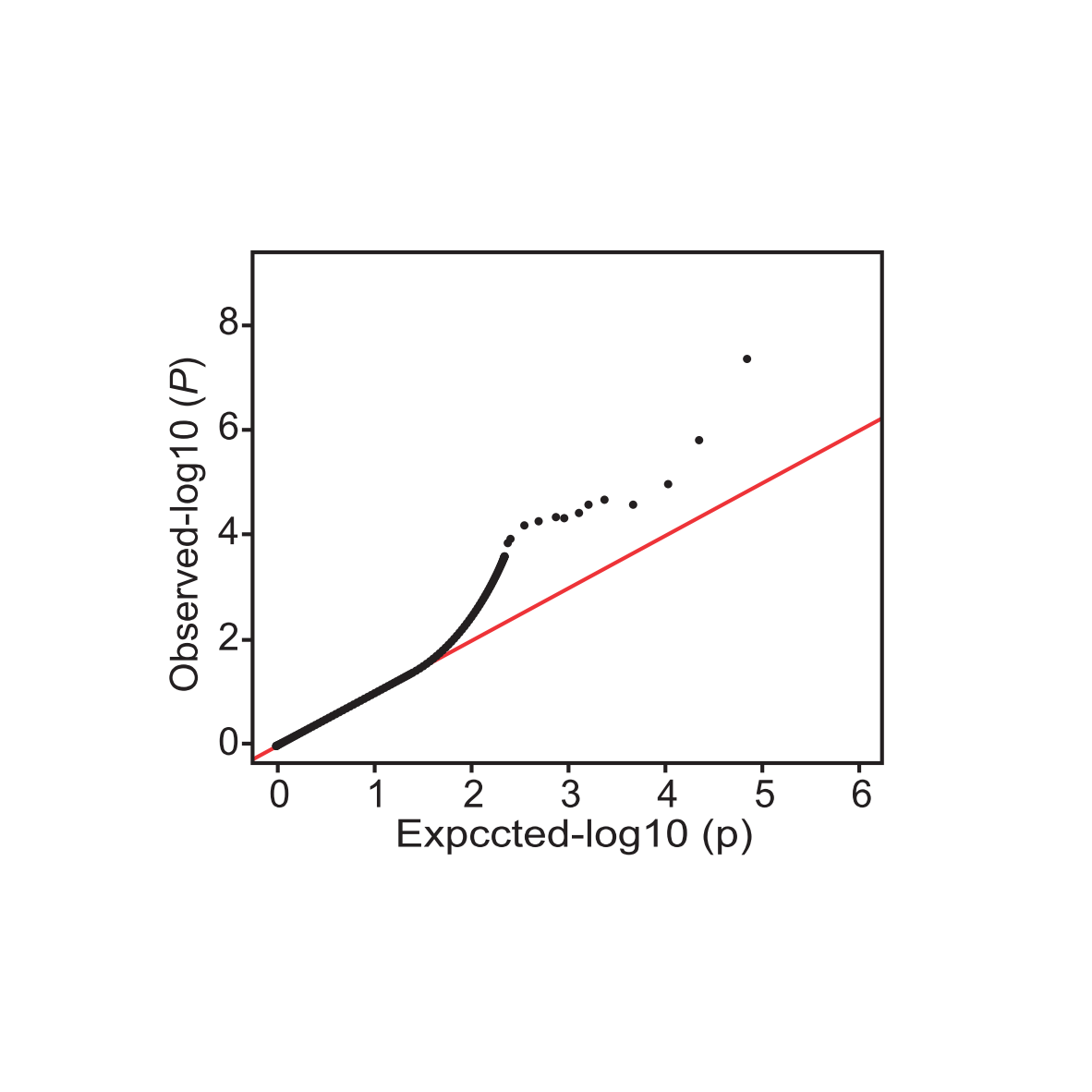

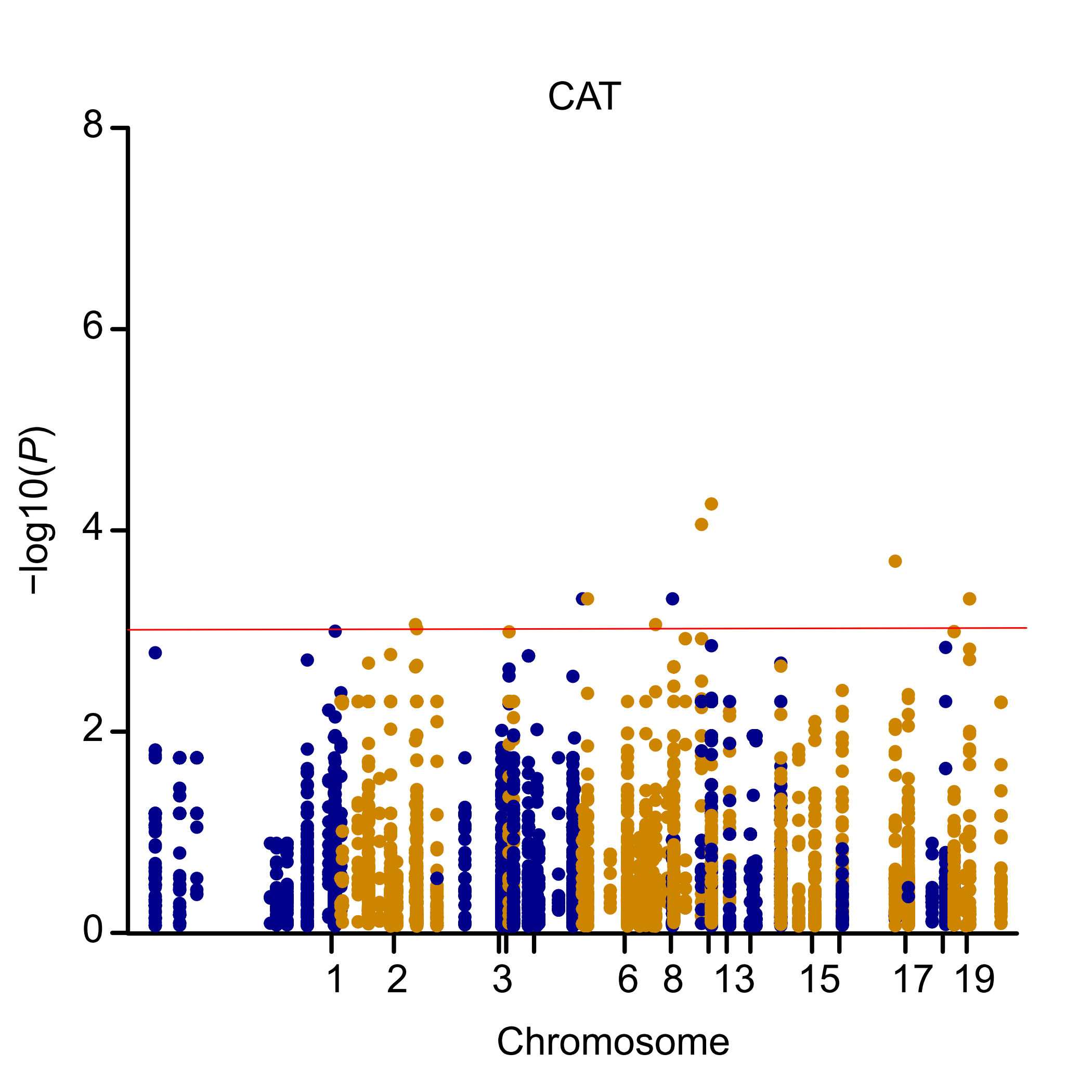

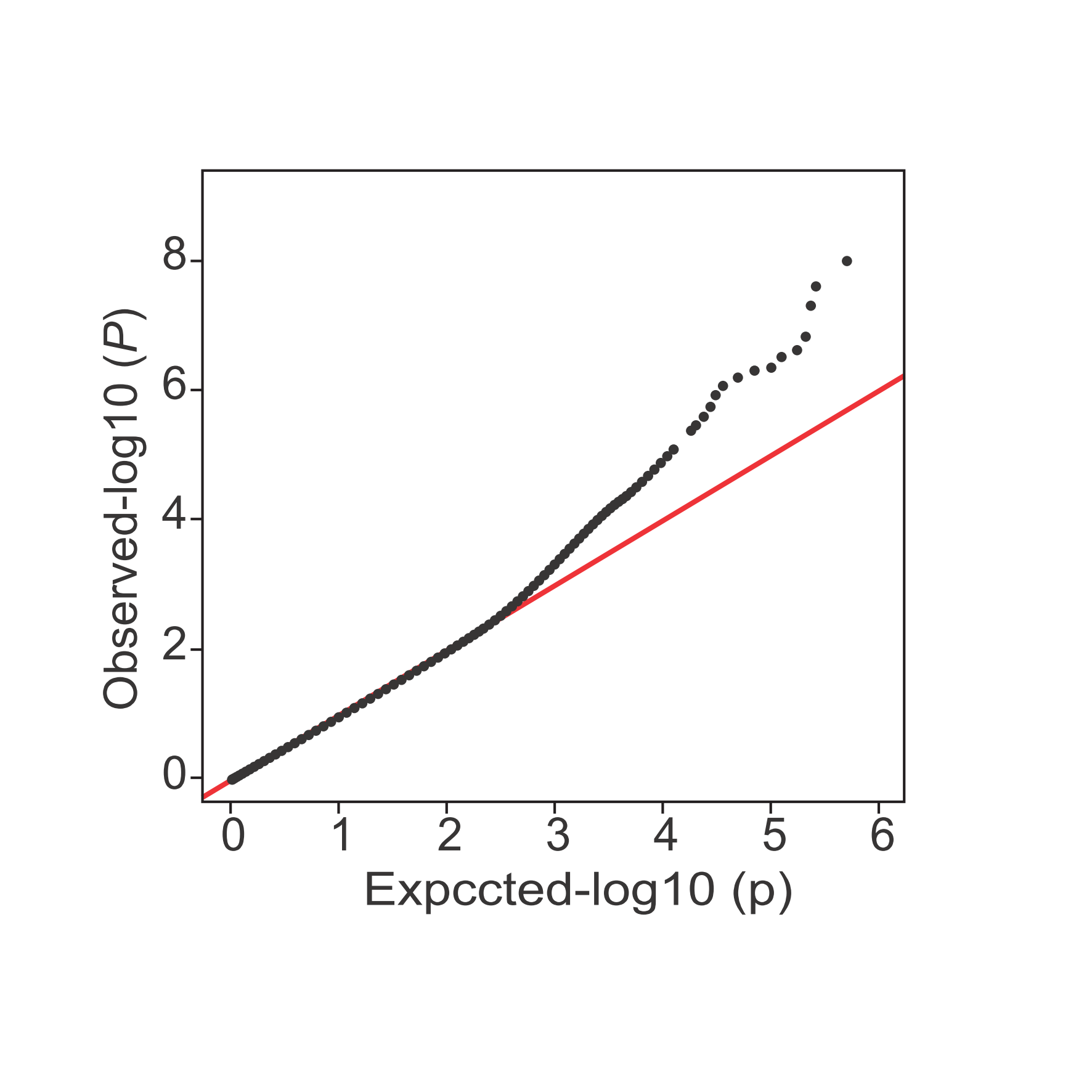


**Figure S4** Manhattan (left) and quantile-quantile plots (right) from the SNP-based association analyses of the six drought stress-related traits in the *P. tomentosa* association population (red line, Bonferroni-adjusted significance threshold [*P* < 0.001]; *x-* and *y-*axes show genomic positions and significances [-log_10_ P-values]; the blue and orange colored dots represent SNPs located on different chromosomes).





**Figure S5** Phylogenetic analysis of *PtoABF3* (A), *PtoLHCA4* (B), *PtoPSB33* (C), and *PtoeIF-2B* (D) in *Populus tomentosa*, 84K (*Populus alba × P. glandulosa*), *Populus trichocarpa*, *Populus euphratica*, *Populus alba*, *Eucalyptus grandis*, *Arabidopsis thaliana*, *Oryza sativa*. The phylogenetic tree was constructed using translated protein sequences.


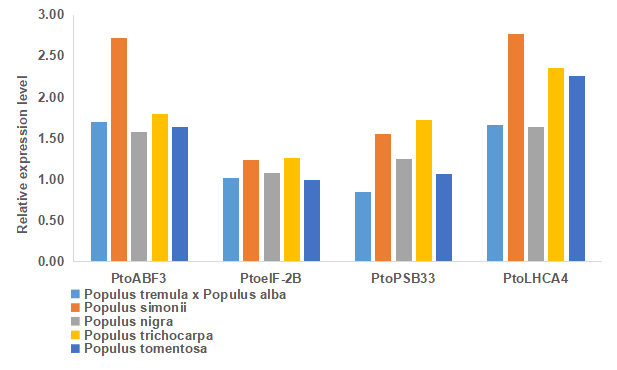


**Figure S6** Expression patterns of the four candidate genes in five poplar species.


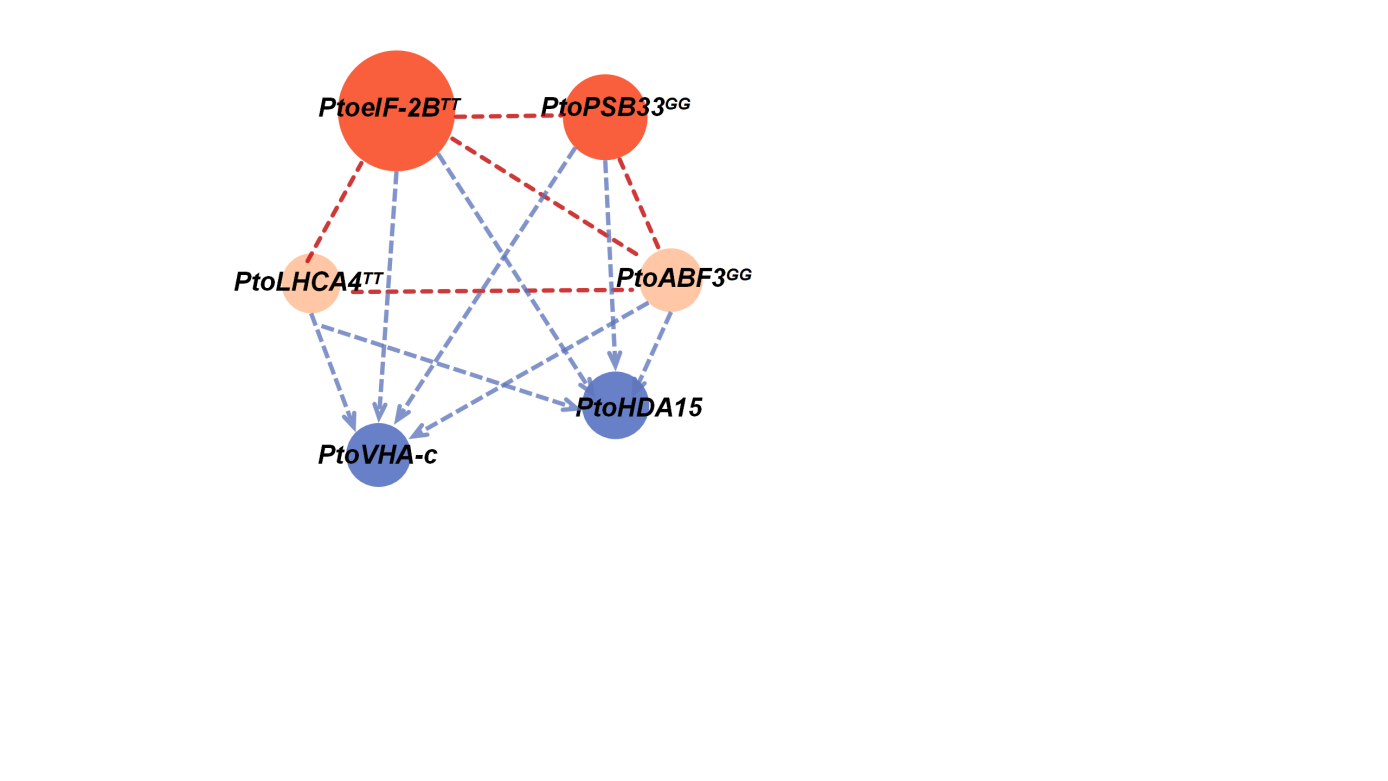


**Figure S7** Allelic genetic regulatory network mediated by four candidate genes involved in drought stress-related traits. *PtoPSB33*, *PtoeIF-2B*, *PtoLHCA4*, and *PtoABF3* were significantly associated with the relative chlorophyll content (Chl) and net photosynthesis (Pn). eQTN mapping showed that *PtoPSB33* and *PtoeIF-2B* acted as the lead trans-eQTNs to mediate the expression patterns of *PtoHDA15* and *PtoVHA-c*, respectively; they indirectly modulated the relative chlorophyll content (Chl). Moreover, *PtoLHCA4* and *PtoABF3* showed epistatic interactions with Chl; they mediated the expression patterns of *PtoHDA15* and *PtoVHA-c*, respectively. Orange and purple circles indicate dominant alleles and candidate genes, respectively. Red lines indicate epistatic interaction; purple dotted arrows indicate *PtoPSB33*, *PtoeIF-2B*, *PtoLHCA4*, and *PtoABF3* mediated the expression patterns of *PtoHDA15* and *PtoVHA-c.* Dotted lines indicate regulatory network according to eQTN mapping and association analyses.
