# Supplementary material for: Transcriptome and association mapping revealed functional genes respond to drought stress in Populus
Source: Front Plant Sci. 2022 Jul 29;13:829888. doi: 10.3389/fpls.2022.829888 (PMC9372527; doi:10.3389/fpls.2022.829888)
Supplement: Supplementary file 1 [file Data_Sheet_1.zip › Supplementary Methods.docx]

### Method S1 Transcriptome data download and processing

The data including *Populus tremula x Populus alba*, *Populus nigra*, *Populus simonii* , *Populus trichocarpa* under different drought conditions was extracted and saved in FASTQ format using the SRA Toolkit, which obtained from the SRA database in NCBI (Gene Expression Omnibus, [http://www.ncbi.nlm.nih.gov/s](http://www.ncbi.nlm.nih.gov/geo)ra). Additionally, ten accessions of *Populus tomentosa* (Ptom) under drought conditions were selected for RNA-seq. Finally, the data sets of 58 samples were collected and provided in the Table S3.

The FASTQ files were first trimmed using Trimomatic software (v.0.32) (Bolger et al., 2014) with the default settings, except for an additional parameter of minimum read length of at least 70% of the original size. Then, the fastq_quality_filter program included in FASTX Toolkit was adopted to further filtrate the FASTQ files, with the minimum quality score 10 and minimum percent of 50% bases that have a quality score larger than this cutoff value. Then the clean data were analysed use the method the same as the ten *Populus tomentosa* above. The unreliable samples and genes were filtered according to the following three criteria: I) The samples, in which the percentage of the number of genes with expression value smaller than 10 reads was larger than 90%, were not considered for further analysis; II) We removed the genes whose expression values were less than 10 reads in more than 80% samples; III) Genes with the variation coefficient of expression values smaller than 0.1 were excluded from subsequent analysis. After filtering, we obtained expression data set composed of 58 RNA-seq samples and 27,640 genes. The transcripts’ expression were normalized based on FPKM.
